# Chronic exposure to synthetic food colorant Allura Red AC promotes susceptibility to experimental colitis via intestinal serotonin in mice

**DOI:** 10.1038/s41467-022-35309-y

**Published:** 2022-12-20

**Authors:** Yun Han Kwon, Suhrid Banskota, Huaqing Wang, Laura Rossi, Jensine A. Grondin, Saad A. Syed, Yeganeh Yousefi, Jonathan D. Schertzer, Katherine M. Morrison, Michael G. Wade, Alison C. Holloway, Michael G. Surette, Gregory R. Steinberg, Waliul I. Khan

**Affiliations:** 1 Department of Pathology and Molecular Medicine, McMaster University, Hamilton, ON Canada; 2 Farncombe Family Digestive Health Research Institute, McMaster University, Hamilton, ON Canada; 3 Department of Biochemistry and Biomedical Sciences, McMaster University, Hamilton, ON Canada; 4 Department of Medicine, McMaster University, Hamilton, ON Canada; 5 Center for Metabolism, Obesity, and Diabetes Research, McMaster University, Hamilton, ON Canada; 6 Department of Pediatrics, McMaster University, Hamilton, ON Canada; 7 Environmental Health, Science and Research Bureau, Health Canada, Ottawa, ON Canada; 8 Department of Obstetrics and Gynecology, McMaster University, Hamilton, ON Canada

**Keywords:** Inflammatory bowel disease, Microbiome

## Abstract

Chemicals in food are widely used leading to significant human exposure. Allura Red AC (AR) is a highly common synthetic colorant; however, little is known about its impact on colitis. Here, we show chronic exposure of AR at a dose found in commonly consumed dietary products exacerbates experimental models of colitis in mice. While intermittent exposure is more akin to a typical human exposure, intermittent exposure to AR in mice for 12 weeks, does not influence susceptibility to colitis. However, exposure to AR during early life primes mice to heightened susceptibility to colitis. In addition, chronic exposure to AR induces mild colitis, which is associated with elevated colonic serotonin (5-hydroxytryptamine; 5-HT) levels and impairment of the epithelial barrier function via myosin light chain kinase (MLCK). Importantly, chronic exposure to AR does not influence colitis susceptibility in mice lacking tryptophan hydroxylase 1 (TPH1), the rate limiting enzyme for 5-HT biosynthesis. Cecal transfer of the perturbed gut microbiota by AR exposure worsens colitis severity in the recipient germ-free (GF) mice. Furthermore, chronic AR exposure elevates colonic 5-HT levels in naïve GF mice. Though it remains unknown whether AR has similar effects in humans, our study reveals that chronic long-term exposure to a common synthetic colorant promotes experimental colitis via colonic 5-HT in gut microbiota-dependent and -independent pathway in mice.

## Abstract (web summary)

Allura Red AC is a dye used in food products. Here the authors report that chronic, long-term exposure to Allura Red AC increases susceptibility to experimental colitis in mice dependent on the serotonin biosynthetic enzyme TPH1, while intermittent exposure more typical for the human setting did not increase susceptibility to experimental colitis.

## Introduction

Environmental factors play an important role in the pathogenesis of various inflammatory and autoimmune diseases^1^. Inflammatory bowel diseases (IBD), Crohn’s disease (CD) and ulcerative colitis (UC), are serious chronic inflammatory diseases of the gastrointestinal (GI) tract; they affect millions of people worldwide^2^. Genetic susceptibility, dysregulated immune response toward perturbed gut microbiota and environmental factors have been shown to contribute to the etiopathogenesis of IBD^3^. Although significant progress has been made to identify susceptible genes and understand the role of the immune system and the gut microbiota, similar advances in defining environmental risk factors for IBD have fallen behind.

There is a growing body of evidence that diet plays a pivotal role in the development of IBD^4,5^. IBD incidence is rising rapidly in developed countries, such as the United States and Canada, and developing countries with a dramatic “westernization” of lifestyle^2^. A western diet is characterized by high intakes of food additives, fats, red meats, and sugar, and low intake of fibers, triggering chronic intestinal inflammation^6–8^. Food additives, such as emulsifiers, stabilizers, and synthetic colorants are widely used to improve the texture, preservation, and aesthetics of processed food. Several studies have reported that high levels of these additives, such as maltodextrin^9^ and titanium dioxide^10^, as well as ingredients added during food processing including dietary emulsifiers (polysorbate-80 and carboxy-methylcellulose^11^) and artificial sweeteners^12^ alter the gut microbiome, increase intestinal permeability, decrease mucus barrier thickness, and promote colitis.

The use of synthetic colorants in dietary products has significantly increased over the past 50 years^13^. These compounds are metabolized to generate free aromatic amines in the gut lumen, some of which are potentially carcinogenic and mutagenic^14,15^. Among many azo dyes, Allura Red AC (FD&C Red 40 or E129) (AR) is the most widely used colorant in many countries^16^ and can be found in commonly consumed dietary products aimed at children (e.g., breakfast cereals, beverages, and confectioneries). AR is a non-genotoxic sulfonated mono-azo red dye that is metabolized by intestinal bacteria through azo reduction^17^. AR exerts pro-inflammatory properties by promoting oxidative stress through reactive oxygen species (ROS) generation and cyclooxygenase-2 (COX-2) expression in the rat liver and kidney^18^. Moreover, 10 mg kg^−1^ of body weight AR administered orally resulted in significant DNA damage in the mouse colon^19^. Despite being highly prevalent in our diet, it is poorly understood how AR influences intestinal inflammation.

Serotonin or 5-hydroxytryptamine (5-HT) is a neurotransmitter and a hormone that regulates GI physiological functions in response to environmental stimuli in the gut. Enterochromaffin (EC) cells are responsible for synthesizing the majority of our body’s 5-HT via the rate-limiting enzyme, tryptophan hydroxylase (TPH) 1; while enteric neurons contribute in small quantities via TPH2^20^. Along with previous findings that 5-HT content and EC cell number were elevated in IBD patients^21–23^, we have previously reported mice with lower 5-HT in the gut as a result of TPH1 knockout (*Tph1*^−/−^) showed reduced colitis severity after dextran sulfate sodium (DSS)^24^. We also found that higher mucosal 5-HT resulted in pro-colitogenic gut microbiota, enhancing susceptibility to colitis^25^. Recently, it was shown *Turicibacter sanguinis* expresses a neurotransmitter sodium symporter-related protein with sequence and structural homology to mammalian serotonin reuptake transporter (SERT)^26^, further supporting a role for 5-HT in shaping the gut microbial composition.

Though some of the azo dyes enhance 5-HT levels in the hypothalamus and brain stem^27^ and modulate 5-HT synthesis in vitro^28,29^, whether there is any interplay among AR, 5-HT and the gut microbiota in influencing colitis susceptibility is unknown. By utilizing experimental models of colitis, intestinal organoids and human cell culture system, we aim to unravel the role of AR in modulating intestinal 5-HT signaling and the gut microbiota in relation to the susceptibility of colitis.

In this work, through a screening of several common synthetic colorants in a model of human EC cells (BON cells), we discover that AR promotes 5-HT secretion. Using mouse models of acute and chronic colitis, we identify chronic, but not the intermittent, exposure for 12 weeks to AR enhances the susceptibility to colitis and that, colonic 5-HT is a key mediator. Higher colonic 5-HT levels and disruption of the epithelial barrier function via myosin light chain kinase (MLCK) by AR exposure are associated with perturbation of the gut microbiota in specific pathogen-free (SPF) mice, while AR exposure also induces mild colitis and elevates colonic 5-HT in naïve germ-free (GF) mice. The ability of AR exposure to induce colitis depends on the bioavailability of colonic 5-HT as evidenced by using mice lacking TPH1 or SERT. Although it remains elusive whether similar effects are observed in humans, our data indicate that chronic exposure to a common synthetic colorant AR promotes experimental colitis via colonic 5-HT in gut microbiota-dependent and -independent pathway in mice.

## Results

### Allura Red AC exacerbates DSS-induced colitis in naïve C57BL/6 mice

As our previous studies revealed a key role of 5-HT in increasing colitis susceptibility^24,25^, we investigated the influence of several common food colorants on the production of 5-HT in BON cells (human EC cell model). Based on their widespread presence in food due to food processing, we selected AR, Brilliant Blue FCF (BB), Sunset Yellow FCF (SY), and Tartrazine Yellow (TY)^30^, and treated BON cells for 24 h with these colorants. All colorants promoted 5-HT secretion and *TPH1* mRNA levels (Supplementary Fig. 1), where AR showed the most pronounced effect at the lowest 1 pmol L^−1^. Based on these findings, we further explored the role of AR as a potential dietary factor in the pathogenesis of colitis.

**Fig. 1:**
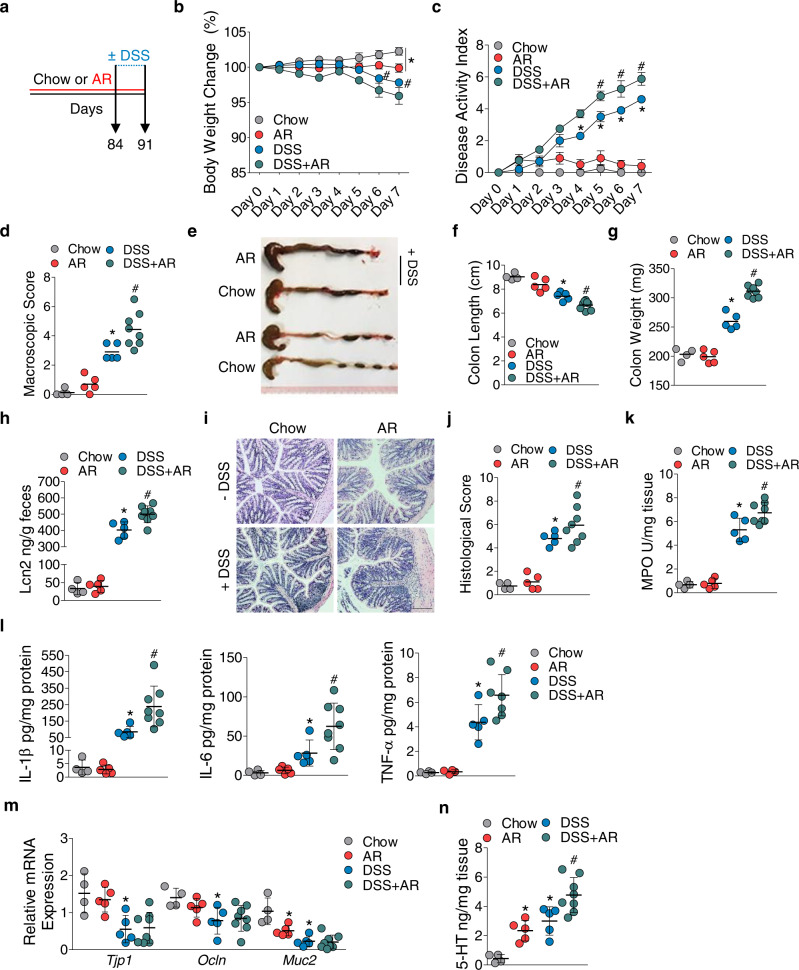
Exacerbation of DSS-induced colitis by AR exposure in C57BL/6 mice. C57BL/6 mice were either fed normal chow diet or exposed to 100 ppm AR via diet for 12 weeks (84 days) prior to induction of acute colitis by 3.5% DSS for 7 days. During DSS, mice were continuously exposed to AR. **a** Schematic illustration of the experimental design. **b** Body weight changes during DSS. **c** Disease activity index (DAI) during DSS. **d** Macroscopic score. **e** A representative image of colons. **f** Colon length (cm). **g** Colon weight (mg). **h** Fecal LCN2 levels. **i** Representative images of hematoxylin and eosin (H&E)-stained colon sections on day 7 post DSS; scale bar: 100 µm. **j** Histological score. **k** Colonic MPO levels. **l** Colonic IL-1β, IL-6, and TNF-α levels. **m** Relative mRNA expression of intestinal epithelial barrier function related genes. **n** Colonic 5-HT level. **b**, **c** Data were analyzed by two-way ANOVA with post hoc Bonferroni’s test and are expressed as mean ± SEM (*n* = 4 for Chow; *n* = 5 for AR and DSS; *n* = 8 for DSS + AR). **d**–**n** Data were analyzed by one-way ANOVA with post hoc Bonferroni’s test and are expressed as mean or mean ± SD (*n* = 4 for Chow; *n* = 5 for AR and DSS; *n* = 8 for DSS + AR). Significance is denoted by *p < 0.05, ^#^*p* < 0.05, where *p < 0.05 versus Chow, and ^#^*p* < 0.05 versus DSS. Source data are provided as a Source Data File.

To understand the effect of AR on colitis development, C57BL/6 mice were either fed a normal chow diet or exposed to AR via diet (100 ppm; a custom diet; TD.190960) for 12 weeks, followed by 3.5% DSS for 7 days. Mice were exposed to AR during DSS while control groups received the control diet (Fig. 1a). AR level was calculated based on the acceptable daily intake (ADI) in humans (7 mg kg^−1^ per body weight) using the previously described formula as a benchmark^12^. Food intake between the groups was not different prior to DSS (Supplementary Fig. 2). Mice exposed to AR without DSS showed reduced body weight, which was further exacerbated in DSS (Fig. 1b). Compared with DSS-treated mice, AR-exposed DSS-treated mice (AR-DSS) showed an increased disease activity index (DAI) (Fig. 1c), which was associated with higher macroscopic scores (Fig. 1d), reduction in colonic lengths (Fig. 1e, f) and increased colonic weights (Fig. 1g). Fecal lipocalin-2 (LCN2) levels were higher in AR-DSS compared to their DSS counterparts (Fig. 1h). Histological scores (Fig. 1i, j) and colonic MPO levels (Fig. 1k) were also higher in AR-DSS compared to DSS-treated mice. In addition, colonic interleukin (IL)−1β, IL-6, and tumor necrosis factor (TNF)-α, were higher in AR-DSS (Fig. 1l) than their DSS counterparts, while the genes that regulate intestinal epithelial barrier function (zonula occludin-1 [ZO-1; *Tjp1*], and occludin [*Ocln*]), were not reduced in AR-DSS compared to their DSS counterparts (Fig. 1m). A significant decrease in *Muc2* mRNA levels was observed in mice exposed to AR without DSS compared to untreated mice (Fig. 1m), suggesting a compromised mucus layer prior to DSS. Furthermore, colonic 5-HT levels were elevated in mice exposed to AR without DSS compared to untreated mice, and on day 7 post DSS compared to their DSS counterparts (Fig. 1n). We also investigated the effect of AR via water in C57BL/6 mice and observed similar results on day 7 post DSS (Supplementary Figs. 3 and 4). These data indicate that AR exacerbates DSS-induced colitis in C57BL/6 mice.

### Allura Red AC triggers an early development of colitis in CD4^+^CD45RB^hi^ T cell-induced colitis model

**Fig. 2:**
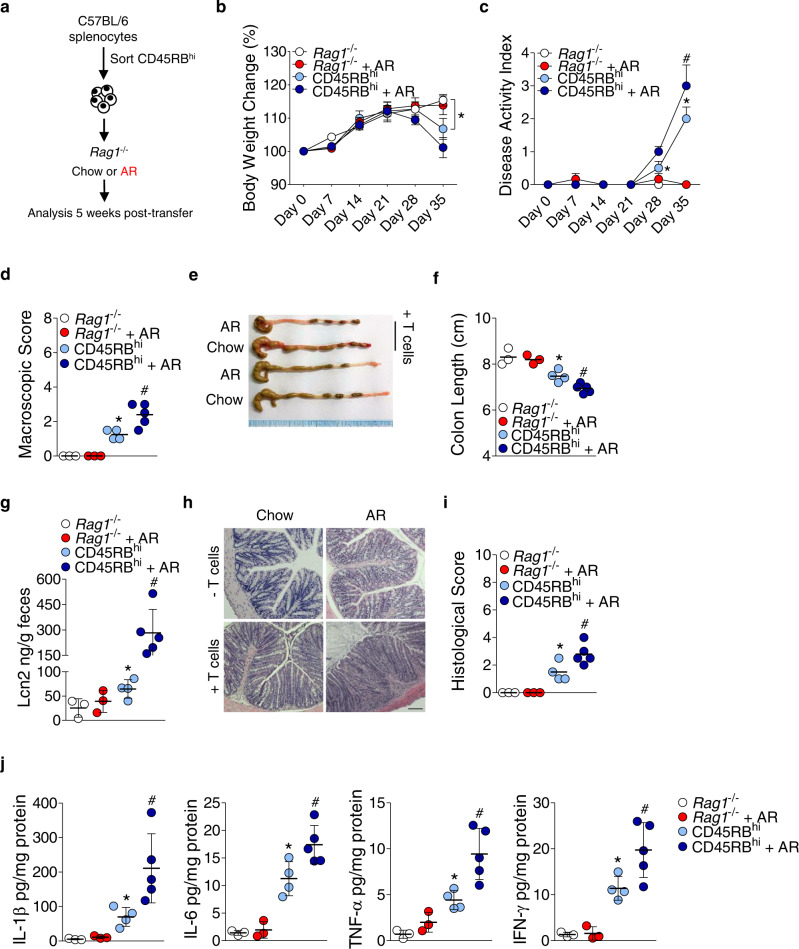
AR exposure exacerbates the development of T cell-induced colitis model. **a** Schematic illustration of the experimental design. Briefly, *Rag1*^−/−^ mice were reconstituted with FACS-sorted CD4^+^CD45RB^hi^ T cells harvested from spleens of healthy C57BL/6 mice. *Rag1*^−/−^ mice were either fed normal chow diet or exposed to 100 ppm AR via diet at the time of reconstitution. **b** Body weight change. **c** Disease activity index (DAI). **d** Macroscopic score. **e** A representative image of colons. **f** Colon length (cm). **g** Fecal LCN2 levels. **h** Representative images of hematoxylin and eosin (H&E)-stained colonic sections; scale bar: 100 µm. **i** Histological score. **j** Colonic IL-1β, IL-6, and TNF-α, and IFN-γ levels. **b**, **c** Data were analyzed by two-way ANOVA with post hoc Bonferroni’s test and are expressed as mean ± SEM (*n* = 3 for *Rag1*^−/−^, and *Rag1*^−/−^ + AR; *n* = 4 for CD45RB^hi^; *n* = 5 for CD45RB^hi^ + AR). **d**–**j** Data were analyzed by one-way ANOVA with post hoc Bonferroni’s test and are expressed as mean or mean ± SD (*n* = 3 for *Rag1*^−/−^, and *Rag1*^−/−^ + AR; *n* = 4 for CD45RB^hi^; *n* = 5 for CD45RB^hi^ + AR). Significance denoted by *p < 0.05, ^#^*p* < 0.05, where *p < 0.05 versus *Rag1*^−/−^, and ^#^*p* < 0.05 versus CD45RB^hi^. Source data are provided as a Source Data File.

To further probe the role of AR in the development of colitis, we used a well-established T cell transfer chronic colitis model. FACS-sorted wild-type (WT) CD4^+^CD45RB^hi^ T cells (Supplementary Fig. 5) were intraperitoneally transferred into *Rag1*^−/−^ mice, and at the time of reconstitution, mice were either fed normal chow diet or exposed to AR via diet for 5 weeks (Fig. 2a). Transfer of T cells induced increased body weight loss (Fig. 2b), increased DAI (Fig. 2c), higher macroscopic score (Fig. 2d), and reduced colonic lengths (Fig. 2e, f) in CD45RB^hi^-AR mice compared to CD45RB^hi^ mice. In addition, fecal LCN2 levels (Fig. 2g) and histological score (Fig. 2h, i) were higher in CD45RB^hi^-AR mice than CD45RB^hi^ mice. Furthermore, colonic IL-1β, IL-6, TNF-α, and interferon [IFN]-γ levels were also increased in CD45RB^hi^-AR mice compared to CD45RB^hi^ mice (Fig. 2j). These findings indicate AR can promote colitis via CD4^+^ T cells in *Rag1*^−/−^ mice.

### Intermittently exposing mice to Allura Red AC does not influence susceptibility to DSS-induced colitis

In the above experiments, C57BL/6 mice were continuously exposed to AR every day for 12 weeks prior to and during DSS. In a separate experiment, C57BL/6 mice were intermittently exposed to AR via diet for one day per week for 12 weeks, followed by 3.5% DSS for 7 days (Supplementary Fig. 6a). Body weight change and DAI were similar in both groups of mice on day 7 post DSS (Supplementary Fig. 6b, c). Macroscopic and histological scores were also not different between the two groups (Supplementary Fig. 6d–f). Moreover, colonic MPO and pro-inflammatory cytokine levels were also not different between the two groups on day 7 post DSS (Supplementary Fig. 6g, h). These data indicate the intermittent exposure of AR for 12 weeks does not enhance the susceptibility to DSS-induced colitis.

### Early life exposure to Allura Red AC enhances susceptibility to DSS-induced colitis

Exposure during early life influences the susceptibility for IBD development in later life^31^. Four-week-old C57BL/6 mice were either fed normal chow diet or exposed to AR via diet for 4 weeks prior to induction of acute colitis with 3.5% DSS for 7 days. To examine whether early life exposure to AR primes mice to enhanced susceptibility to DSS-induced colitis, mice were not exposed to AR during DSS (Supplementary Fig. 7a). Mice exposed to AR showed increased body weight loss compared to their DSS counterparts on day 7 post DSS (Supplementary Fig. 7b). Compared with DSS counterparts, DSS-treated mice, which were exposed to AR, showed higher DAI (Supplementary Fig. 7c). This increased DAI was accompanied by higher macroscopic scores (Supplementary Fig. 7d) and reduction in colonic length (Supplementary Fig. 7e, f). Histological scores (Supplementary Fig. 7g, h) and colonic MPO levels (Supplementary Fig. 7i) were also higher in mice exposed to AR compared to their DSS counterparts on day 7 post DSS. In addition, colonic 5-HT levels were elevated in mice exposed to AR without DSS compared to untreated mice, and this increase was further substantiated on day 7 post DSS (Supplementary Fig. 7j). Moreover, AR potently increased colonic IL-1β, IL-6, and TNF-α levels in mice on day 7 post DSS compared to their DSS counterparts (Supplementary Fig. 7k). These data indicate early life exposure to AR primes mice to heightened susceptibility to DSS-induced colitis.

### Allura Red AC induces low-grade colonic inflammation in naïve C57BL/6 mice

**Fig. 3:**
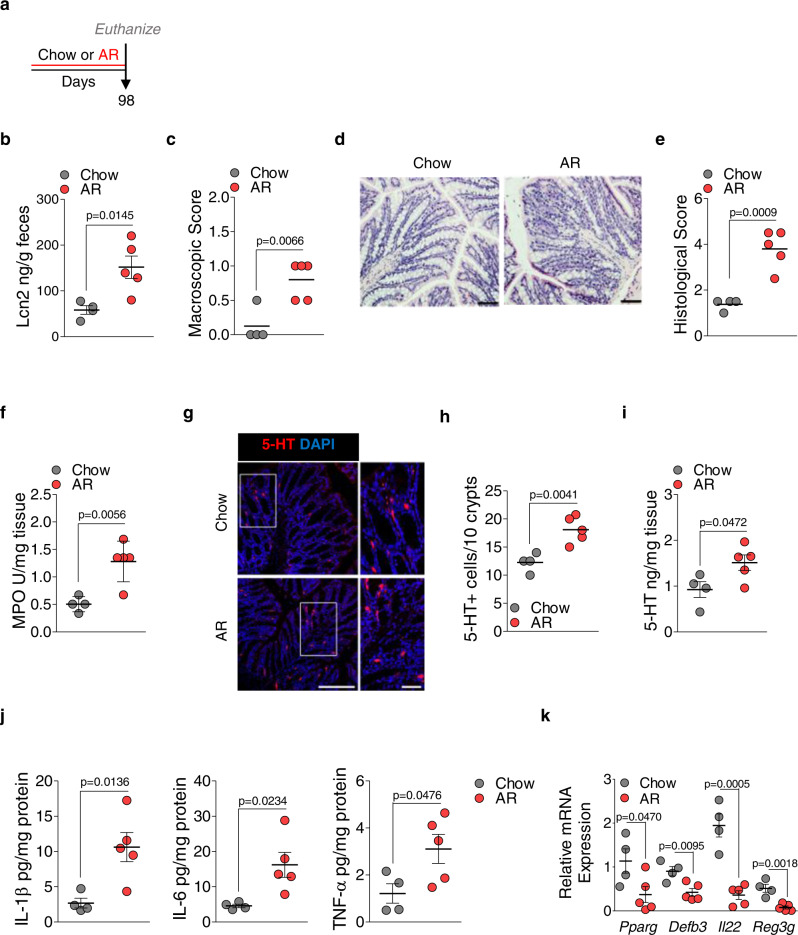
AR promotes low-grade colonic inflammation in naïve C57BL/6 mice. Naïve C57BL/6 mice were either fed normal chow diet or exposed to 100 ppm AR via diet for 14 weeks (98 days). **a** Schematic illustration of the experimental design **b** Fecal LCN2 levels. **c** Macroscopic score. **d** Representative images of H&E-stained colon sections; scale bar: 50 µm. **e** Histological score. **f** Colonic MPO levels. **g** Representative images of immunofluorescence (IF) staining for 5-HT (red) and nuclei by DAPI (blue) in the colon sections; scale bar: 50 µm. **h** Number of 5-HT^+^ cells per 10 crypts. **i** Colonic 5-HT level. **j** Colonic IL-1β, IL-6, TNF-α levels. **k** *Pparg*, *Defb3*, *Il22*, and *Reg3g* mRNA levels. **b**–**k** Data were analyzed by two-tailed unpaired Student’s test and are expressed as mean or mean ± SD (*n* = 4 for Chow; *n* = 5 for AR). Source data are provided as a Source Data File.

Body weight (Fig. 1b) and *Muc2* mRNA levels were reduced (Fig. 1m), while colonic 5-HT levels were increased (Fig. 1n), in mice exposed to AR without DSS. These observations led us to examine whether AR promotes low-grade colonic inflammation without DSS (Fig. 3a). Mice were exposed to AR for 14 weeks (7 days more than the exposure in Fig. 1), and food intake was not different between the groups (Supplementary Fig. 8). Mice exposed to AR showed 4.41 ± 1.96 ng mL^−1^ of AR concentration in the serum and higher fecal LCN2 levels than control (Fig. 3b). These mice also showed higher macroscopic (Fig. 3c) and histological scores (Fig. 3d, e), and colonic MPO levels (Fig. 3f) than control mice. The number of 5-HT^+^ cells was also increased in the colon of AR-exposed mice (Fig. 3g, h), along with increased colonic 5-HT levels (Fig. 3i) and colonic IL-1β, IL-6, and TNF-α levels (Fig. 3j) than control mice. Moreover, genes related to antimicrobial responses, such as peroxisome proliferator-activated receptor gamma (*Pparg*), β-defensin 3 (*Defb3*), IL22 (*Il22*), and regenerating islet-derived protein (REG) 3 gamma (*Reg3g*), were downregulated in the colon of mice exposed to AR compared to control mice (Fig. 3k).

**Fig. 4:**
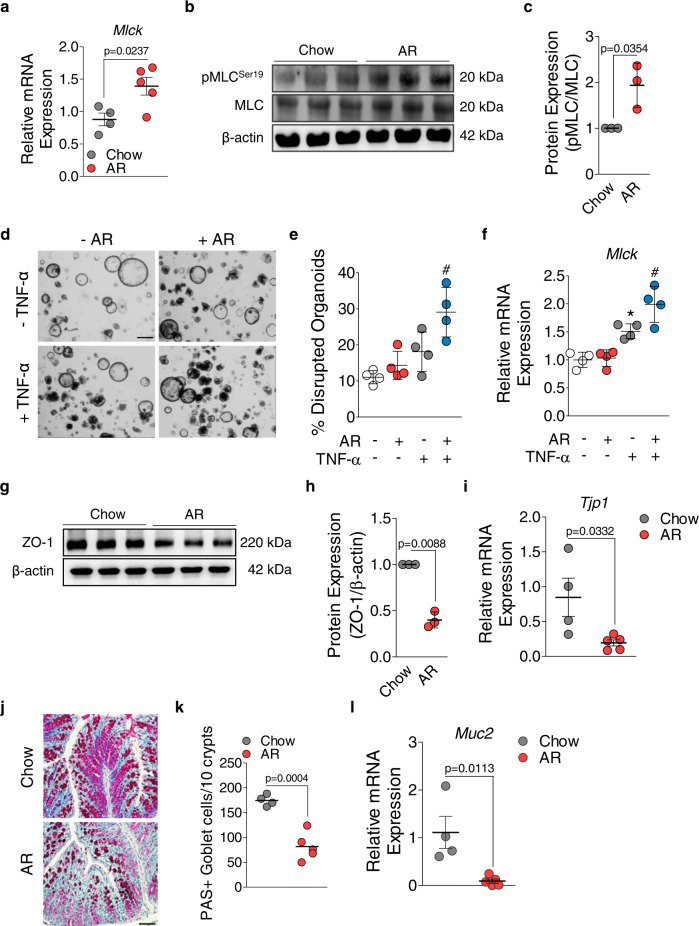
AR activates MLCK signaling pathway and alters intestinal epithelial barrier function in naïve C57BL/6 mice and murine intestinal organoids. **a** *Mlck* mRNA levels. **b** Representative western blot analysis of pMLC^Ser19^, MLC, and β-actin. Uncropped blots are provided in the Source Data. **c** Relative densitometry (pMLC/MLC) (*n* = 3 mice/group). **d** Representative bright field (BF) images of mouse colonic organoids treated for 24 h with or without AR (1 μmol L^−1^) following pre-treatment for 1 h with or without TNF-α (10 ng/mL). **e** Percentage of disrupted organoids. **f** *Mlck* mRNA levels in 2D monolayer derived from murine colonic organoids. **g** Representative western blot analysis of ZO-1 and β-actin. Uncropped blots are provided in the Source Data. **h** Relative densitometry (ZO-1/β-actin) (*n* = 3 per group). **i** *Tjp1* mRNA levels. **j** Representative images of PAS–stained colon sections; scale bar: 50 µm. **k** Number of PAS^+^ colonic goblet cells per 10 crypts. **l** *Muc2* mRNA levels. **a**–**l** (except **e** and **f**) Data were analyzed by two-tailed unpaired Student’s *t*-test and are expressed as mean or mean ± SD (*n* = 4 for Chow; *n* = 5 for AR). **e**, **f** Data were analyzed by one-way ANOVA with post hoc Bonferroni’s test. Data are expressed as mean ± SD and representative of 2 independent experiments. Significance denoted by *p < 0.05, ^#^*p* < 0.05 unless otherwise provided, where *p < 0.05 versus untreated 2D monolayer derived from murine colonic organoids, and ^#^*p* < 0.05 versus TNF-α treated 2D monolayer derived from murine colonic organoids. Source data are provided as a Source Data File.

To elucidate the underlying mechanism by which AR alters the intestinal epithelial barrier function, we cultured HT-29 cells to first examine whether the epithelial cells respond to AR. The cytochrome P450 (CYP) family 1 enzymes are mainly controlled by aryl hydrocarbon receptor (AhR), which is involved in phase I xenobiotic metabolism in the intestine^32^. Induction of CYP1A1 and CYP1B1 enhances inflammatory responses and alters the intestinal epithelial barrier function^33,34^. Consistent with previous findings that azo dyes can induce CYP1 enzymes^35^, AhR was activated (Supplementary Fig. 9a, b) and both *CYP1A1* and *CYP1B1* mRNA levels were increased after 24 h of AR treatment (1 μmol L^−1^) (Supplementary Fig. 9c, d). The myosin light chain kinase (MLCK) pathway regulates intestinal epithelial barrier function, and MLCK is necessary for TNF-α induced barrier loss by phosphorylating myosin II regulatory light chain (MLC)^36^. MLCK-dependent regulation has also been shown in inflamed colonic tissues of IBD patients and mice with colitis, in which increased MLCK phosphorylated MLC protein at Serine 19^37^. We observed higher *Mlck* mRNA levels (Fig. 4a) and increased pMLC^Ser19^ in the colon of AR-exposed mice (Fig. 4b, c). The levels of both *MLCK* mRNA and pMLC^Ser19^ were also increased when HT-29 cells were pre-treated for 1 h with TNF-α (10 ng mL^−1^), followed by 24 h of AR treatment (1 µmol L^−1^) (Supplementary Fig. 9e–g).

To mimic a more physiological microenvironment, we performed ex vivo culture of murine colon-derived crypt organoids. Prior to treatment with AR (1 µmol L^−1^), murine colonic organoids were exposed to TNF-α (10 ng mL^−1^)^38^. When organoids were exposed to AR for 24 h following 1 h of TNF-α treatment, a reduction in the relative number of organoids with altered morphology was observed, while untreated organoids showed an intact columnar morphology (Fig. 4d, e). As a sphere-like geometry prevents access to the apical side of the epithelium, we obtained 2D monolayers derived from mouse 3D organoids as described previously^39^. In a functional 2D monolayer evidenced by accumulated ZO-1 at the apical intercellular membrane (Supplementary Fig. 10), we observed no difference in *Mlck* mRNA levels when the monolayers were treated with AR for 24 h, compared to untreated (Fig. 4f). However, when the monolayers were pre-treated for 1 h with TNF-α prior to AR exposure for 24 h, a significant upregulation of *Mlck* mRNA levels was observed compared to TNF-α (Fig. 4f). As MLCK activation and subsequent phosphorylation of MLC^Ser19^ was observed, we measured ZO-1 and *Tjp1* mRNA levels, which were significantly decreased in mice exposed to AR (Fig. 4g–i). Protective mucus layers generated by goblet cells are necessary for maintaining a healthy intestinal mucosal barrier, and the impairment of which correlates with increased microbiota-induced colitis^40^. Depletion of colonic PAS^+^ cell numbers and lower *Muc2* mRNA expression levels were observed in mice exposed to AR compared to control (Fig. 4j–l). Together, these data indicate AR exposure impairs the intestinal epithelial barrier function by activating MLCK pathway and induces low-grade colonic inflammation in the absence of DSS.

### Colonic serotonin plays an important role in enhancing colitis severity by Allura Red AC

**Fig. 5:**
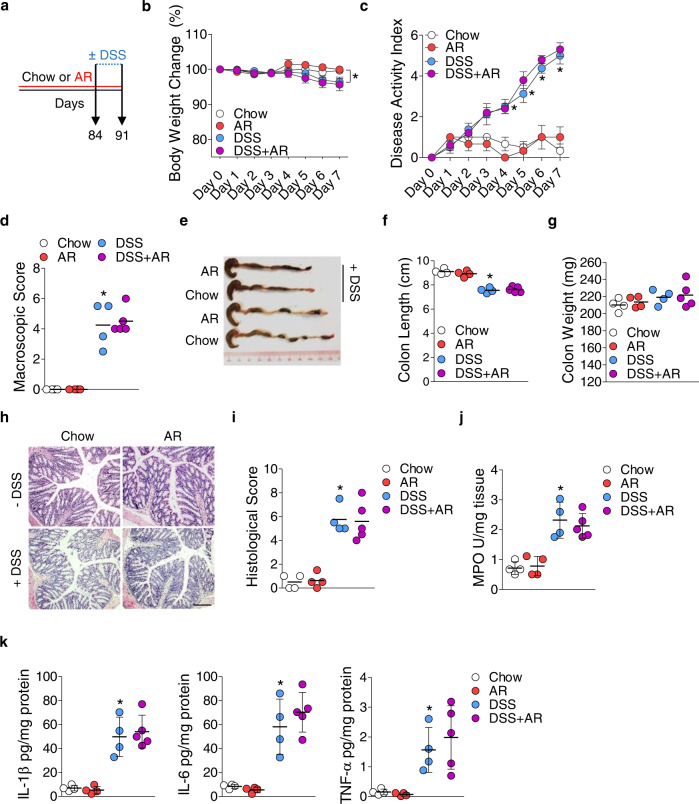
AR does not exacerbate the development of DSS-induced colitis in TPH1-deficient mice. *Tph1*^−/−^ mice were either fed normal chow diet or exposed to 100 ppm AR via diet for 12 weeks (84 days) prior to induction of acute colitis by 3.5% DSS for 7 days. During DSS, mice were continuously exposed to AR. **a** Schematic illustration of the experimental design. **b** Body weight changes during DSS. **c** Disease activity index (DAI) during DSS. **d** Macroscopic score. **e** A representative image of colons. **f** Colon length (cm). **g** Colon weight (mg). **h** Representative images of H&E-stained colon sections on day 7 post DSS; scale bar: 100 µm. **i** Histological score. **j** Colonic MPO levels. **k** Colonic IL-1β, IL-6, and TNF-α levels. **b**, **c** Data were analyzed by two-way ANOVA with post hoc Bonferroni’s test and are expressed as mean ± SEM (*n* = 4 for Chow, AR, and DSS; *n* = 5 for DSS + AR). **d**–**k** Data were analyzed by one-way ANOVA with post hoc Bonferroni’s test and are expressed as mean or mean ± SD (*n* = 4 for Chow, AR, and DSS; *n* = 5 for DSS + AR). Significance is denoted by ^*^p < 0.05, ^#^*p* < 0.05, where *p < 0.05 versus Chow, and ^#^*p* < 0.05 versus DSS. Source data are provided as a Source Data File.

Higher colonic 5-HT levels were associated with AR-induced colitis. To delineate the role of 5-HT in mediating the effect of AR, *Tph1*^−/−^ mice were either fed normal chow diet or exposed to AR via diet for 12 weeks, followed by 3.5% DSS for 7 days (Fig. 5a). Mice were continuously exposed to AR during DSS. We observed similar food intake (Supplementary Fig. 11) and colitis assessment, namely body weight change, DAI, macroscopic score, colon length, and colon weight, between DSS-treated and AR-exposed DSS-treated mice (Fig. 5b–g). Histological scores (Fig. 5h, i) and colonic MPO levels (Fig. 5j) were also similar between the two groups. In addition, colonic pro-inflammatory cytokine levels were not different between DSS groups (Fig. 5k). Likewise, when *Tph1*^−/−^ mice were exposed to AR via water, there was no difference in DSS-induced colitis (Supplementary Figs. 12 and 13).

To further substantiate the intermediary effect of 5-HT in AR-induced colitis, SERT-deficient (SERT^−/−^) mice, which exhibit enhanced DSS-colitis severity due to augmented bioactivity of 5-HT^41^ were exposed to AR via diet for 12 weeks prior to induction of acute colitis with 3.5% DSS for 7 days (Supplementary Fig. 14a). SERT^−/−^ mice exposed to AR showed decreased body weight and elevated DAI compared to control on day 7 post DSS (Supplementary Fig. 14b, c). The increased DAI was accompanied by higher macroscopic and histological scores (Supplementary Fig. 14d–f). Higher colonic MPO (Supplementary Fig. 14g) and 5-HT levels (Supplementary Fig. 14h) were observed in SERT^−/−^ mice exposed to AR along with increased colonic IL-1β, IL-6, and TNF-α levels (Supplementary Fig. 14i) on day 7 post DSS compared to their DSS counterparts.

We next elucidated the underlying mechanism by which AR promotes 5-HT secretion using BON cell. ROS is a well-characterized driver of colitis pathogenesis^42^ and NF-κB activity is regulated by intracellular ROS level^43^. We observed an elevation of intracellular ROS level detected by 2’,7’-dichlorofluorescein diacetate (DCF-DA) fluorescence after 24 h of AR treatment (Supplementary Fig. 15a). However, we did not observe any effect of the AR-derived metabolite, *p*-Cresidinesulfonic acid in modulating 5-HT biosynthesis and ROS level (Supplementary Fig. 15b–d). To further investigate the relationship between AR and 5-HT, we pre-treated BON cells for 1 h with triptolide (20 nmol L^−1^), a potent NF-κB inhibitor, followed by 24 h of AR treatment. Triptolide attenuated the effect of AR on *TPH1* mRNA levels and 5-HT secretion (Supplementary Fig. 15e, f). Similarly, 5-HT secretion was increased when the monolayer derived from mouse colonic organoids was treated for 24 h with AR (Supplementary Fig. 15g). Collectively, these data indicate AR directly promotes 5-HT secretion from EC cells by increasing ROS level and activating NF-κB, while AR does not increase the susceptibility to DSS in the absence of TPH1 in vivo.

### Perturbed gut microbiota by Allura Red AC enhances colitis susceptibility

**Fig. 6:**
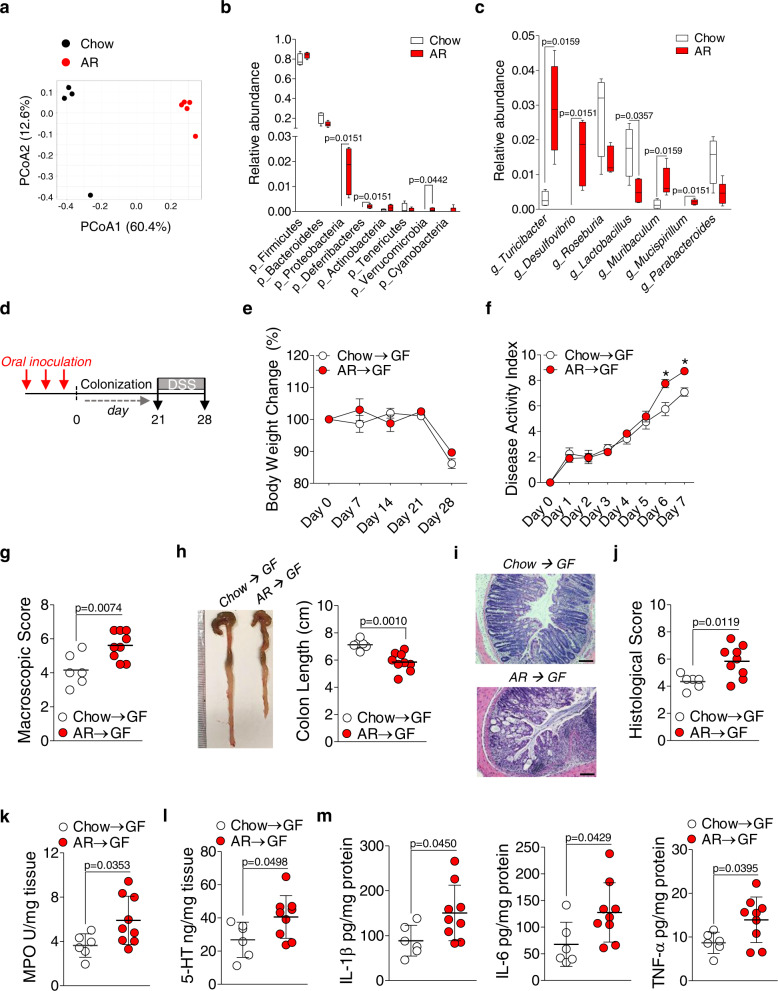
Transplantation of altered gut microbiome from AR-exposed mice into GF mice exacerbates DSS-induced colitis. **a**–**c** 16S rRNA bacterial profiling at the v3–v4 region using cecal contents was carried out. **a** Bray–Curtis dissimilarity revealed each group of mice possessed distinct microbiota. Each dot represents an individual mouse; *n* = 4 (Chow) and *n* = 5 (AR). Box-and-whisker plots illustrate the median, quartiles, maximum and minimum of relative abundance of bacteria **b** phylum and **c** genus level. **b** and **c** Whiskers are from the 10–90 percentile with a median in the center, and data were analyzed by two-tailed Mann–Whitney *U* test (*n* = 4 for Chow; *n* = 5 for AR). **d**–**m** Cecal contents were pooled at equal amounts from SPF C57BL/6 mice either fed normal chow diet or exposed to 100 ppm AR via diet. A 200 μL inoculum was administered to each group of GF mice orally once per day for 3 consecutive days, followed by 21 days of colonization prior to induction of acute colitis with 2.0% DSS for 7 days. All recipient GF mice fed normal chow diet during experiment. **d** Schematic illustration of the experimental. Red arrow indicates daily gavage. **e** Body weight change from day 0 to day 28 (where DSS was given during the last 7 days). **f** Disease activity index (DAI) during DSS. **g** Macroscopic score. **h** Colon length (cm). A representative image of colons. **i** Representative images of H&E-stained colon sections on day 7 post DSS; scale bar: 100 μm. **j** Histological score. **k** Colonic MPO levels. **l** Colonic 5-HT levels. **m** Colonic IL-1β, IL-6, and TNF-α levels. **e**, **f** Data were analyzed by two-way ANOVA with post hoc Bonferroni’s test and are expressed as mean ± SEM (*n* = 6 for Chow → GF; *n* = 9 for AR → GF). **g**–**m** Data were analyzed by two-tailed unpaired Student’s *t*-test and are expressed as mean or mean ± SD (*n* = 6 for Chow → GF; *n* = 9 for AR → GF). Significance is denoted by *p < 0.05 unless otherwise provided, where *p < 0.05 versus Chow → GF. Source data are provided as a Source Data File.

We next examined whether the gut microbiota has any role in increasing susceptibility to colitis in AR-exposed C57BL/6 mice. Bacterial profiling in cecal contents was performed to elucidate whether low-grade colonic inflammation observed in C57BL/6 mice (see Figs. 3 and 4) was associated with changes in the gut microbiota composition. Analysis of β-diversity using Bray–Curtis dissimilarity revealed a markedly distinct clustering pattern between the two groups on the PCoA1 axis (Fig. 6a) along with changes in the relative abundance at the phylum and genus level (Fig. 6b, c); neither *Escherichia* nor *Klebsiella* was detected. These findings suggest that increased colitis susceptibility is associated with an altered microbial profile.

To substantiate the observation that perturbed gut microbiota by AR exposure is associated with increased colitis susceptibility, GF mice were orally inoculated with an equally weighted pool of cecal contents derived from mice exposed to AR or control for 3 days (once per day), followed by 21 days of colonization prior to 2.0% DSS for 7 days (Fig. 6d). During the experiment, all mice received normal chow diet and the transplanted microbiota profiles persisted during 21 days of colonization (Supplementary Fig. 16). GF mice that received cecal contents from AR-exposed mice (GF-AR) showed increased severity of colitis compared to GF mice that received cecal contents from control mice (GF-NC). Although the body weight change was similar between the two groups (Fig. 6e), DAI was significantly higher in GF-AR mice (Fig. 6f), which was associated with higher macroscopic score (Fig. 6g) and shortened colonic lengths (Fig. 6h), compared to GF-NC mice. In addition, histological score (Fig. 6i, j) and colonic MPO levels (Fig. 6k) were elevated, along with increased colonic 5-HT levels in GF-AR mice (Fig. 6l), compared to GF-NC mice. Moreover, colonic IL-1β, IL-6, and TNF-α levels were elevated in GF-AR mice compared to GF-NC mice (Fig. 6m). These data indicate that perturbed gut microbiota induced by AR exposure exacerbates the severity of DSS-induced colitis.

**Fig. 7:**
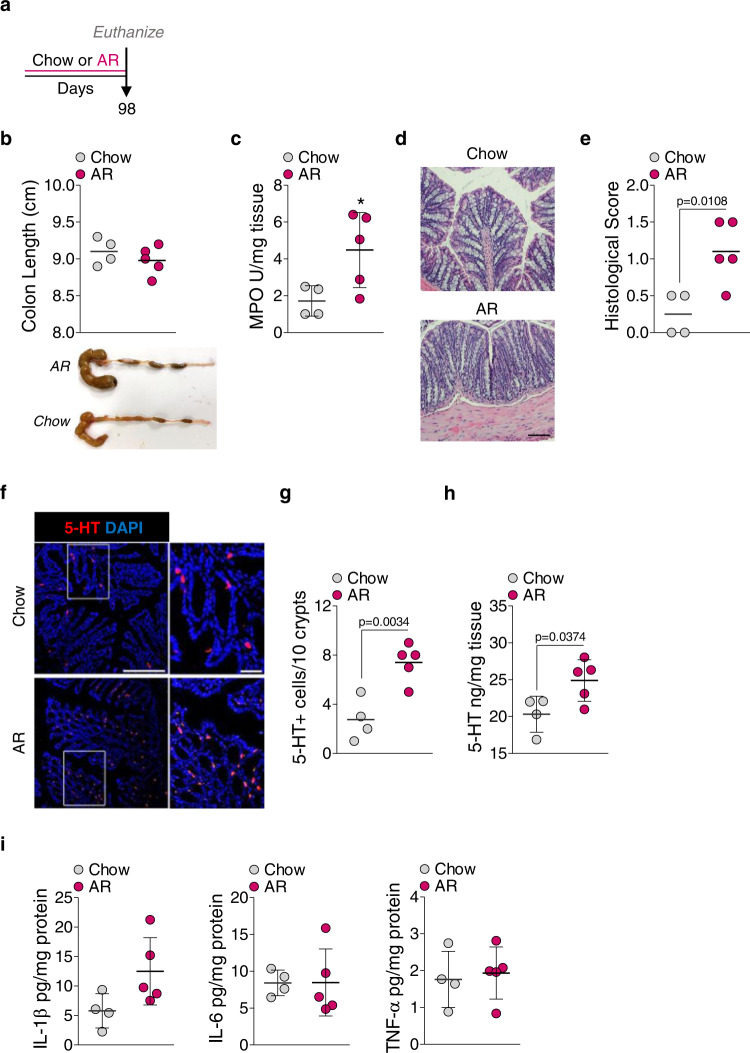
AR increases colonic 5-HT levels in the absence of microbial signals. GF mice were either fed normal chow diet or exposed to 100 ppm AR via diet for 14 weeks (98 days). **a** Schematic illustration of the experimental design. **b** Colon length (cm). A representative image of colons. **c** Colonic MPO levels. **d** Representative images of H&E-stained colon sections; scale bar: 100 μm. **e** Histological score. **f** Representative images of IF staining for 5-HT (red) and nuclei by DAPI (blue) in the colon; scale bar: 50 µm. **g** Number of 5-HT^+^ cells per 10 crypts. **h** Colonic 5-HT levels. **i** Colonic IL-1β, IL-6, and TNF-α levels. **b**–**i** Data were analyzed by two-tailed unpaired Student’s *t*-test and are expressed as mean or mean ± SD (*n* = 4 for Chow; *n* = 5 for AR). *p < 0.05 versus Chow. Source data are provided as a Source Data File.

To further discern whether increased colitis susceptibility by AR exposure is mediated by the gut microbiota, GF mice were either fed normal chow diet or exposed to AR via diet for 14 weeks (Fig. 7a). Similar colonic lengths were observed (Fig. 7b). However, colonic MPO levels (Fig. 7c) and histological scores (Fig. 7d, e) were higher in mice exposed to AR compared to control. In addition, the number of 5-HT^+^ cells was increased (Fig. 7f, g), along with higher colonic 5-HT levels in mice exposed to AR (Fig. 7h), compared to control. Colonic pro-inflammatory cytokines, such as IL-1β, IL-6, and TNF-α, were not different (Fig. 7i). These findings indicate AR promotes 5-HT synthesis and inflammatory signals via both microbiota-dependent and -independent pathways.

## Discussion

**Fig. 8:**
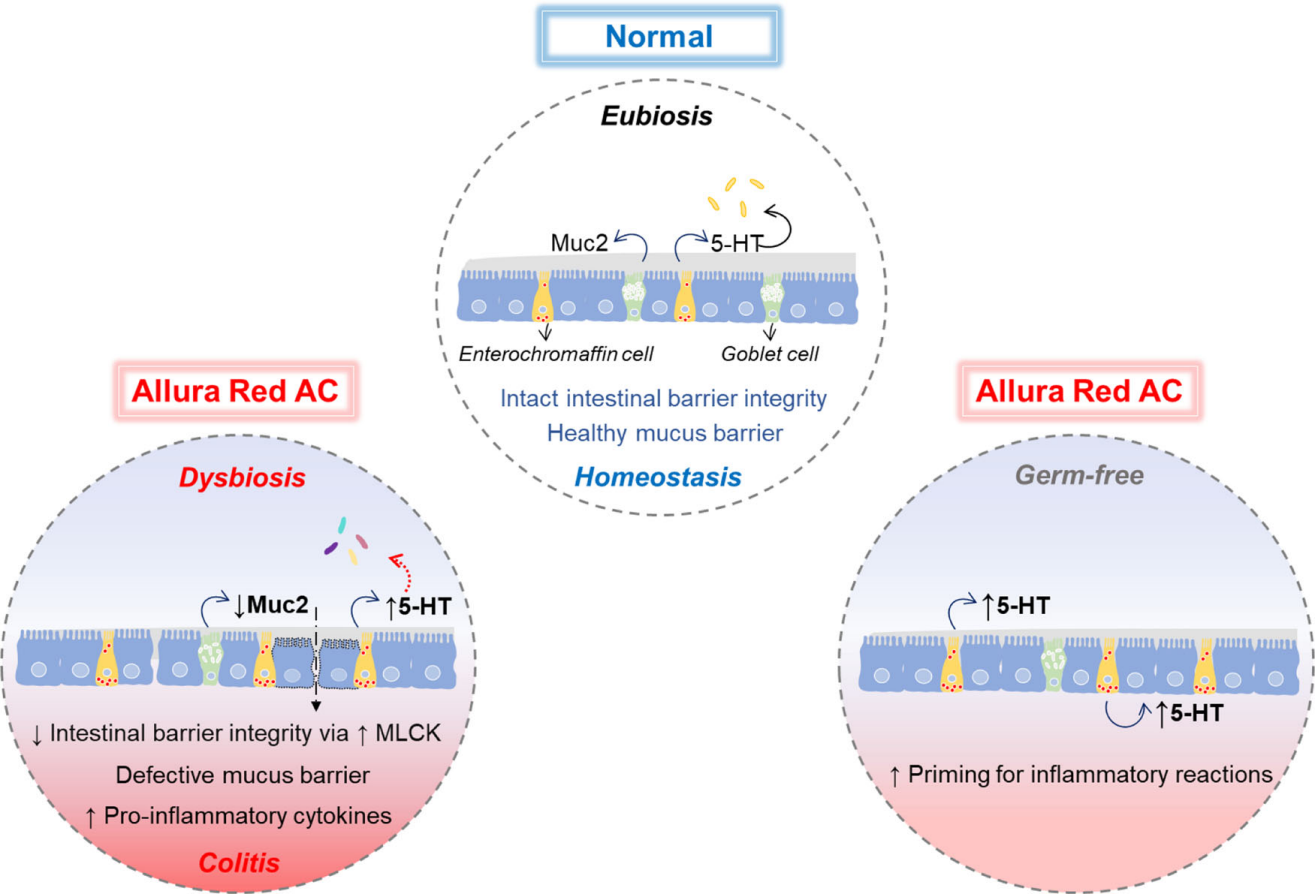
Graphical summary of AR effects on the development of colitis. Graphical abstract showing the effect of AR in the development of colitis. AR disrupts the intestinal epithelial barrier integrity via MLCK and mucus layer, while AR also stimulates colonic 5-HT secretion, modulates the gut microbiota composition, and promotes colitis. Under GF conditions, AR also induces colonic 5-HT secretion, which primes for inflammatory reactions.

Humans are exposed to various chemical substances everyday through diet. Western diets are especially rich in synthetic colorants that enhance the appearance of foods to attract consumers, particularly children. Although several dietary risk factors that are associated with chronic diseases were identified^9–11^, our knowledge on the role of these dietary components on IBD pathogenesis is still modest. Here, we provide evidence that a widely used synthetic colorant AR enhances colitis susceptibility via colonic 5-HT in gut microbiota-dependent and -independent pathway under healthy conditions in mice (Fig. 8).

Our studies revealed that AR exacerbated DSS-induced colitis and induced low-grade colonic inflammation. Impairment of the intestinal epithelial barrier function via MLCK was associated with colitis. In addition, our findings indicate colonic inflammation by AR exposure was associated with higher colonic 5-HT levels. It has previously shown that 5-HT directly downregulates the intestinal epithelial barrier proteins^44^, suggesting that increased 5-HT levels may exacerbate the effects of AR exposure in the impairment of the epithelial barrier function.

Persistent low-grade inflammation is a common underlying feature of many chronic diseases^45^, and MLCK activation is associated with increased epithelial permeability^46^. In addition, increased MLCK correlated with clinical activity in IBD patients^37^, whereas MLCK-mediated tight junction dysregulation in intestinal epithelium led to apoptosis-mediated barrier loss and the induction of experimental colitis^47^. These findings are further consistent with previous studies where the loss of intestinal epithelial barrier mediated by hyperpermeability precedes the onset of colitis in IBD patients and their clinically healthy first-degree relatives of those with CD or in individuals with familial risk^48–50^.

*Tph1*^−/−^ mice maintain normal 5-HT level in the brain and neuronal tissues due to the sustained presence of TPH2 isoform^51^, thus exhibiting no differences in behavior and GI physiological functions^52^. Dysregulation of intestinal 5-HT, however, leads to pathological consequences in the gut. IBD patients show increased EC cell numbers and 5-HT levels in both plasma and serum along with reduced SERT expression in the mucosa^22,23^. Experimental models of colitis have shown that increased mucosal 5-HT signaling promotes intestinal inflammation^24,53,54^. Our data illustrated a connection between 5-HT, AR exposure and inflammation. This is supported by our findings that showed AR exposure did not influence susceptibility to colitis in *Tph1*^−/−^ mice, while AR increased 5-HT and elevated the number of 5-HT^+^cells in the colon of C57BL/6 mice. These findings are in agreement with the importance of TPH1-derived 5-HT in the pathogenesis of colitis, which is supported by previous findings that pharmacologically inhibiting mucosal 5-HT synthesis uncouple the positive linkage of colonic 5-HT to colitis^55,56^. Moreover, SERT-deficient mice show increased severity of experimental models of colitis due to augmented bioactivity of 5-HT^41,54^. In this study, we observed SERT^−/−^ mice exposed to AR showed increased severity of DSS-colitis along with higher colonic 5-HT levels on day 7 post DSS compared to SERT^−/−^ mice that were not exposed to AR. Furthermore, when GF mice were exposed to AR, colonic 5-HT levels were increased. Together, these findings illustrate that any direct effects of AR on the gut microbiota do not influence colitis susceptibility if TPH1-derived 5-HT production is inhibited, suggesting that the indirect effect of AR via the host’s serotonergic system on the gut microbiota contributes to a greater extent.

Similarly, an increased 5-HT secretion via NF-κB through direct induction of ROS generation by AR suggests a positive feedback cycle is in play and further drives 5-HT biosynthesis. Given that high 5-HT levels induce NADPH oxidase (NOX2)-derived ROS and primes colonic epithelial cells toward inflammation^44^, it is reasonable to state that AR exposure primes colonic epithelium toward inflammatory responses through 5-HT. In addition to directly promoting inflammation, there is a complex relationship between 5-HT and the gut microbiome^57^. Expansion of *Turicibacter* by AR exposure was associated with higher colonic 5-HT levels. This observation can be supported by a previous study that found *T*. *sanguinis* imports 5-HT and grows to higher levels by competing with surrounding microbes and signaling nearby EC cells through a mechanism that is inhibited by selective serotonin reuptake inhibitor (SSRI)^26^. As mice with depleted CD8^+^ T cells have increased *Turicibacter*^58^, and that is related to increased TNF-α^59^, this suggests that *Tph1*^−/−^ mice may be protected from AR-induced colitis in part by altering the microbiome. Further investigation is warranted to precisely understand the role of *Turicibacter* in colitis.

It has previously shown that the immunopathogenic mechanisms induced by AR at a dosage, which is lower than those considered safe in humans (0.25 mg mL^−1^), in transgenic mice overexpressing IL-23 were dependent on IFN-γ-producing CD4^+^ T cell-mediated responses^60,61^. The occasional exposure in these studies was sufficient to induce colitis in the primed mice as genome-wide association studies (GWAS) in humans link the IL-23 signaling pathway with IBD^62^ and therapies targeting IL-23 are effective in IBD patients^63^. In our studies, however, when C57BL/6 mice were intermittently exposed to AR (24 h per week) for 12 weeks before DSS-induced colitis, there was no difference in the colitis severity. It appears that the exposure level in our experimental design was not sufficient to promote colitis compared to the level being exposed every day. Future studies with a longer period of AR exposure would be important for further understanding of the effect of intermittent exposure on colitis.

The current data suggest that AR enhances the susceptibility to colitis. It should be mentioned that the effect of the AR’s microbially derived metabolite, CS, was not further tested in vivo as CS did not promote 5-HT secretion in vitro. This finding is consistent with previous study that found CS did not induce colitis in mice^60^. However, AR has been observed to inhibit the growth of certain gut microbiota, and even in the absence of growth inhibition, AR can induce broad changes in bacterial gene expression (Syed, personal communication, 2022). Furthermore, it has previously been postulated that bacterial azoreductase activity can be saturated at a high concentration of AR, which would ultimately lead to the accumulation of the parent compound in the lumen even in the presence of azo dye-metabolizing strains^17^. The possibility of saturation of the enzyme activity by the chronic administration of the dye and subsequent reduction in decolorization rate of the dye cannot be excluded.

Our findings that the exposure of AR during early life primes mice to the heightened susceptibility to DSS-induced colitis support the notion that early life is increasingly considered as a crucial period that influences susceptibility to IBD development in later life^31^. This is particularly important since synthetic colorants are a convenient and low-cost alternative for food manufacturers to make foods even brighter and more appealing to the customer, particularly young children. As food colorants are prevalent in many foods commonly consumed by children, they may have greater exposure than adults. Our study investigated the cumulative effect of AR, which is relevant to individuals regularly consuming (intentionally or unintentionally) foods rich in synthetic colorants. In this study, exposing mice to a single dosage is the limitation, which requires further studies with multiple doses of AR. In addition, the current dosage may not recapitulate, to some extent, the average exposure humans encounter in daily life. Thus, population-based studies on AR consumption by humans including IBD patients are necessary to ascertain if the intake of synthetic food colorants is associated with later development of colitis. As dietary products contain multiple colorants, investigating whether any synergistic actions between these colorants influence the susceptibility to colitis will be a key direction for future work.

Collectively, we show that chronic, but not the intermittent exposure to AR enhances colitis susceptibility, and that colonic 5-HT is a key mediator for the observed phenotype in mice. As our knowledge of the associations between perturbed gut microbiota and IBD expands, the effects of environmental factors, such as diet, are increasingly becoming a public health concern. Our findings provide important data on the potential role of AR in promoting murine colitis and warrant further investigations on the roles of other commonly used food dyes, such as BB, SY, and TY, in the pathogenesis of colitis. Moreover, future studies are necessary to identify similar effects in humans. This study thus will not only prompt scrutiny of its use in many industries but also advance public awareness to prevent adverse health consequences.

## Methods

### Animals

C57BL/6N (Taconic), C57BL/6J (Jackson Laboratory), *Rag1*^−/−^ (Jackson Laboratory) and SERT^−/−^ (Jackson Laboratory) were kept in sterilized, filter-topped cages under SPF conditions. Germ-free (GF; in-house C57BL/6 background) mice were bred under GF conditions in the Axenic Gnotobioic Unit (AGU) at McMaster University. Breeding pairs of *Tph1*^−/−^ and *Tph1*^+/+^ mice were obtained from CNRS, Paris, France, and were kept and bred under SPF conditions. All mice were housed in sterilized filter-topped cages with free access to autoclaved normal chow food and water at a temperature of 21–22 °C, and with 12:12 h light/dark cycle in an SPF room of McMaster University Central Animal Facility. All mice were acclimatized for 10 days prior to the start of any experiments. Male and female mice aged 8–12 weeks were used. Allura red AC was purchased from Toronto Research Chemical. Mice received either a control diet (Teklad Irradiated Global 18% Protein Rodent Diet, 2918) or a custom diet (a control diet coated with Allura Red AC (0.1 g kg^−1^; TD.190960, Teklad). Allura Red AC at 0.01% w/v (0.1 mg mL^−1^) solution was also used for experiments using drinking water. Animal welfare was reviewed during the application of the animal experiments by the Central Animal Facility, and all animal experiments were carried out in accordance with the McMaster University animal utilization protocols and with approval from the McMaster University Animal Care Committee and McMaster Animal Research Ethics Board (AREB). Animal welfare was monitored during the experiments by the employees of the Central Animal Facility of McMaster University, and all mice were euthanized by manual cervical dislocations.

### DSS-induced colitis

DSS (molecular mass 40 kDa; ICN; Biomedicals Incorporate, Solon, OH) was added to drinking water for a final concentration of 2.0% and 3.5% w/v for a total of 7 days. All experiments were approved by the McMaster University animal ethics committee and conducted under the Canadian guidelines for animal research. Samples from all mice were kept at −80 °C.

### T cell-induced colitis

C57BL/6 and *Rag1*^−/−^ mice were purchased from Jackson Laboratory. All mice were kept in sterilized, ventilated cages under SPF conditions and fed either a control diet or exposed to AR via the diet. Mice were allowed to acclimatize for 7 days prior to the start of experiments. Colitis was induced in *Rag1*^−/−^ mice by adoptive transfer of FACS-sorted CD4^+^CD45RB^hi^ T cells. Naïve CD4^+^ T cells were isolated from splenocytes of C57BL/6 mice by EasySep^TM^ Mouse Naïve CD4^+^ T cell Isolation Kit (StemCell Technology, Vancouver, Canada). Naïve CD4^+^ T cells were labeled with PE-cy7-conjugated anti-mouse CD3 (BioLegend; 1:100), APC-conjugated anti-mouse CD4 (BD Biosciences; 1:100), and FITC-conjugated anti-mouse CD45 (BD Biosciences; 1:100). CD4^+^CD45RB^hi^ T cells were sorted using FACS Aria II flow cytometer (BD Biosciences; FACSDiva v 6.1.2). Cell viability was assessed using Trypan blue assay prior to injection. Recipients were intraperitoneally (i.p.) injected with 5 × 10^5^ cells of sorted T cells. Samples from all mice were kept at −80 °C.

### Assessment of colitis severity

Disease activity index (DAI) is a combined score of weight loss, stool consistency, and fecal bleeding, and was blindly assessed^24^. The scoring system was defined as follows: weight loss: 0 = no loss, 1 = 1–5%, 2 = 5–10%, 3 = 10–20%, 4 = 20%+; stool: 0 = normal, 2 = loose stool, 4 = diarrhea; and bleeding: 0 = no blood, 2 = Hemoccult positive (Hemoccult II; Beckman Coulter, Fullerton, CA), and 4 = gross blood (blood around anus). DAI was measured on all 7 days of DSS treatment. Macroscopic damage scores were blindly scored using a previously published scoring system for DSS-induced colitis^24^. The severity of colitis was macroscopically scored based on colonic bleeding, fecal bleeding, loosening of stool consistency, and signs of rectal bleeding.

### Histology and immunohistochemistry

Colons were washed with PBS, fixed in 10% buffered formalin, washed with ethanol, and embedded in paraffin. H&E-stained colonic tissue sections are scored for the following measures^24^: crypt architecture (normal, 0 to severe crypt distortion with loss of entire crypts, 3), degree of inflammatory cell infiltration (normal, 0 to dense inflammatory infiltrate, 3), muscle thickening (base of crypt sits on the muscularis mucosae, 0 to marked muscle thickening present, 3), goblet cell depletion (absent, 0 to present, 1) and crypt abscess (absent, 0 to present, 1). The histological damage score is the sum of each individual score. Formalin-fixed, paraffin-embedded sections of intestines were stained with periodic acid-Schiff (PAS) stain to detect intestinal goblet cells. The number of PAS^+^ goblet cells was expressed per 10 crypts. Investigators were blinded to the study groups. Images were captured using a Nikon Eclipse 80i microscope and NIS-Elements Basic Research imaging software (v 3.2).

### Cecal microbiota transfer

Cecal contents from each group of mice were pooled (equally weighed) and GF mice were orally administered with 200 µL (diluted in phosphate buffer saline [PBS]) for 3 days with a single dose each day.

### Measurement of myeloperoxidase (MPO) level

Colonic MPO levels were measured. Colonic tissue samples (1 cm of mid-distal colon) were weighed after removing any visible feces or fat.

Hexadecyltrimethylammonium bromide (HTAB) buffer (Sigma) was added according to tissue weight. If tissue weight is less than 25 mg, add the buffer at a ratio of 12.5 mg mL^−1^; if tissue weight is between 25 and 50 mg, add at a ratio of 25 mg mL^−1^. Colonic tissues in ice-cold 50 mM potassium phosphate buffer containing 0.5% HTAB were then homogenized using a Mixer Mill (MM 400, Retsch, Inc., Newtown, PA) at 30 frequency (1 s^−1^) for 4 min. Homogenates were centrifuged 13,400 × g for 6 min and the supernatant was removed, and each sample (7 µL) was then added to a 96-well plate, and 200 µL of solution containing potassium phosphate buffer, *O*-dianisidine (Sigma), and hydrogen peroxide. The absorbance was measured at 450 nm by a spectrophotometer (model EL808, BioTek, Winooski, VT). MPO levels were expressed in units per milligram of wet tissue, where 1 U is the quantity of enzyme able to convert 1 μmol hydrogen peroxide to water in 1 min at room temperature.

### Cell culture

Human colonic adenocarcinoma HT-29 cells (ATCC HTB-38; kindly gifted from Dr. Kris Chadee from the University of Calgary, Canada) were maintained in Dulbecco modified Eagle medium (DMEM)/F12 (1:1) with 10% (v/v) heat-inactivated fetal bovine serum (FBS), supplemented with modified Eagle medium and HEPES buffer (pH 7.5) as well as penicillin/streptomycin at 37 °C in a humidified 5% CO_2_ atmosphere. HT-29 cells were seeded in a 24-well culture plate at a density of 10^5^ cells mL^−1^. Cells were allowed to attach for overnight, which were then replenished with the serum-free media. Cells were stimulated with Allura Red AC (1 µmol L^−1^), indole-3-carboxyaldehyde (I3A) (50 or 500 µmol L^−1^), TNF-α (10 ng mL^−1^) or medium alone for 24 h. BON cells (human carcinoid cell line derived from a metastasis of a pancreatic carcinoid tumor of EC cell origin; kindly gifted by Dr. Dusan Bogunovic from Icahn School of Medicine at Sinai Health) were cultured in DMEM/F12 (1:1) supplemented with 10% FBS and 1% penicillin/streptomycin. Briefly, cells were seeded at 10^5^ cells mL^−1^ in a 24-well plate and incubated for 24 h at 37 °C in the complete growth medium. The medium was replaced with serum-free media prior to treatment. Cells were treated with Allura Red AC (1 µmol L^−1^), 5-HT (10 µmol L^−1^) for 24 h or pre-treated for 1 h with triptolide prior to Allura Red AC treatment (20 nmol L^−1^; Tocris Biosciences, Burlington, Canada) for 24 h. Following the treatment, the cell supernatants and RNA were collected and stored at −80 °C for further analysis. Intracellular ROS production detected by 2’,7’-dichlorofluorescein diacetate (DCF-DA) fluorescence as previously described^44^. Briefly, cells were treated with Allura Red AC (1 µmol L^−1^) or 5-HT (10 µmol L^−1^) for 24 h, followed by DCF-DA treatment at a final concentration of 10 µmol L^−1^ at 37 °C for 30 min. Cells were then washed twice with PBS. Images were captured by a Nikon Eclipse 80i microscope and NIS-Elements Basic Research imaging software (v 3.2). Investigators were blinded to the study groups.

### Generation of mouse colon organoids and maintenance

Murine colonoids were isolated based on previously published methods^39,64^. Briefly, whole colon was washed with cold PBS, and fats were removed. Colon was then cut longitudinally (or butterfly) to remove feces and washed with cold PBS. Colon was gently scraped off to remove villi, mucus, and other debris using forceps. Next, colon was vortexed 10 times with ice-cold advanced DMEM/F12 with replacing the medium to the new medium after each wash. After the last wash, colon was placed in 5 mL of cell recovery solution (Cat #: 354253; Corning, Corning, NY) at 4 °C for 1 h. Then, colon was centrifuged twice at 1500 rpm for 5 min, followed by being diluted 1:1 in Matrigel (Corning). After the Matrigel was solidified, organoid media (10 mL of base media: Advanced DMEM/F12, supplemented with penicillin/streptomycin [100 U mL^−1^], GlutaMAX [100X], and HEPES [0.01 mol L^−1^]) with 10 mL of 50% WRN supplemented with N2 (100X) (Invitrogen, Burlington, Canada), B27 (Invitrogen), N-acetylcystine (1 mmol L^−1^) (Sigma-Aldrich), nicotinamide (Sigma-Aldrich), mouse epidermal growth factor (mEGF; 50 ng mL^−1^) (Invitrogen), A83-01 (500 nmol L^−1^) (Tocris Biosciences), SB-202190 (10 µmol L^−l^) (Sigma-Aldrich), Y-27632 (10 µmol L^−1^) (Abmole Biosciences, Houston, TX) were added prior to incubation at 37 °C with 5% CO_2_ atmosphere. The medium in each well was changed every 3 days. During this time, the complete medium did not contain Y-27632. The colonoids were passaged every 7 days. Upon each passage, Y-27632 was added.

### Colonoid-derived monolayer seeding and stimulation

Monolayers derived from colonoids were generated as previously described^39^. Briefly, the growth media was removed, and Matrigel domes were mechanistically disrupted with gentle pipetting through a p200 pipette tip. Colonoids were resuspended in 150 µL TrypLE express (Gibco, Mississauga, Canada) and incubated at 37 °C with 5% CO_2_ for 7.5 min. Colonoids were then rapidly disrupted into single-cell suspensions with gentle pipetting through a p1000 tip, and an equal volume of monolayer media (base media supplemented with 50% WRN, N2 (Invitrogen), B27 (Invitrogen), mEGF (Invitrogen) and Y-27632 (Abmole) was added. Cells were centrifuged at 1500 rpm for 5 min, then resuspended in monolayer media and added dropwise to Geltrex (Gibco) coated coverslips in 12- or 24-well plates. Coverslips were coated with Geltrex and maintained at 37 °C with 5% CO_2_ atmosphere for 24–48 h prior to the mechanical disruption of Matrigel domes. Monolayers were incubated at 37 °C with 5% CO_2_ and the medium was changed to the monolayer medium without Y-27632 the next day. After 5 days of culture, monolayers were pre-treated for 1 h with or without TNF-α (10 ng mL^−1^), followed by 24 h with or without Allura Red AC (1 μmol L^−1^).

### Quantitative real-time polymerase chain reaction

Colonic tissues, mouse colonic organoids, monolayer derived from organoids, or cell from cell lines were processed with Trizol reagent (Invitrogen, Burlington, Canada) using a Mixer Mill (MM 400, Retsch, Inc., Newtown, PA) at 30 frequency (1 s^−1^) for 5 min. Total RNA was quantified using a NanoDrop One/OneC Microvolume UV-Vis Spectrophotometer (Thermo Fisher Scientific, Mississauga, Canada). RNA purity was assessed by the ratio of absorbance at 260/280 nm, and only samples with a ratio of ~2.0 for RNA were considered. Complementary DNA (cDNA) was prepared from 1 μg of total RNA using iScript cDNA Synthesis Kit (Bio-Rad Laboratories, Mississauga, Canada). Relative quantitative RT-PCR amplification was performed using SsoFast EvaGreen Supermix (Bio-Rad Laboratories, Mississauga, Canada) with the primers at a concentration of 10 μmol L^−1^. 18S was used as an internal standard. Data were analyzed according to the 2^−ΔΔCT^ method. Primer sequences used in the study are listed in Supplementary Tables 1 and 2.

### Enzyme-linked immunosorbent assay (ELISA)

Colon tissue 5-HT levels were measured by using commercially available enzyme-linked immunosorbent assay (ELISA) kits (Cat. # IM1749; Beckman Coulter, Fullerton, CA). Briefly, a small section of colon was weighed and homogenized in 0.2 N perchloric acid. After centrifugation at 10,000 × g for 5 min, the supernatants were collected, and the pH was neutralized by using 1 mol L^−1^ borate buffer. The supernatants were used for the analysis of 5-HT levels using a commercially available ELISA kit (Cat. # IM1749; Beckman Coulter, Fullerton, CA). 5-HT content was expressed as a function of tissue weight (ng mg^−1^). For measurement of 5-HT level in culture supernatants from BON cells and mouse colon organoids, the supernatants were collected and directly measured using the kit. For colon tissue cytokines measurement, a small section of colon was homogenized in Tris-buffered saline containing a protease inhibitor mixture (Cat. # P8340; Sigma-Aldrich, Oakville, Canada). Samples were centrifuged for 5 min at 3300 × g, and the resulting supernatants were frozen at –80 °C until use. Total protein levels were quantified in the colon homogenates by using DC Protein Assay Kit (Cat. # 5000111; Bio-Rad Laboratories). Cytokine levels (IL-1β, Cat. # SMLB00C; IL-6, Cat. # SM6000B; IFN-γ, Cat. # MIF00; and TNF-α, Cat. # MTA00B) were determined according to the manufacturer’s instructions (Quantikine Murine; R&D Systems, Minneapolis, MN).

### Western blot

Western blot was performed as described previously^65^. Briefly, total protein of tissue homogenates or cell lysates was extracted using radio-immunoprecipitation assay (RIPA) buffer containing a 1× protease inhibitor cocktail (PIC). Protein concentration of homogenized tissue was determined by using DC Protein Assay Kit (Bio-Rad Laboratories). Equal amounts of protein homogenates from each group were loaded and electrophoresed onto sodium dodecyl sulfate (SDS)–polyacrylamide gel electrophoresis and transferred to a polyvinylidene difluoride (PVDF) membrane. Membranes were blocked with 5% bovine serum albumin (BSA) in 1× TBST for 1 h at room temperature and incubated with primary antibodies against pMLC^Ser19^ (1:1000) (Cat # 3671; Cell Signaling Technology), total MLC (1:1000) (Cat # 3672; Cell Signaling Technology), ZO-1 (1:1000) (Cat # 40-2200; Thermo Fisher Scientific), and AhR (1:1000) (Cat # sc-133088; Santa Cruz Biotechnology) for overnight at 4 °C. Membranes were washed, incubated with either anti-rabbit horseradish peroxidase–linked antibody (1:5000, Cat. # 7074; Cell Signaling Technology) or anti-mouse horseradish peroxidase–linked antibody (1:5000, Cat. # 7076; Cell Signaling Technology) for 1 h at room temperature. Proteins were visualized by use of SuperSignal West Femto Maximum Sensitivity Substrate (Thermo Fisher Scientific). β-actin (1:1000) (Cat # 4970; Cell Signaling Technology) or Lamin B1 (1:1000) (Cat # ab65986, Abcam, Cambridge, MA) was used as a loading control. Densitometric analysis was performed on western blots with ImageJ software (version 1.48), normalized to total MLC, β-actin, or Lamin B.

### Immunofluorescence staining

For colonic tissue immunostaining, coverslips were deparaffinized at 60 °C for 1 h in xylenes using three changes for 5 min each, followed by washing through graded alcohols: wash in 100% ethanol twice for 10 min each, then 95% ethanol twice for 10 min each. Coverslips were then washed with dH_2_O and blocked with 0.01% Triton X-100 and 0.05% Tween 20 for 1 h at room temperature. Next, coverslips were stained with anti-mouse 5-HT antibody (1:100; Cat. # ab16007, Abcam, Cambridge, MA) overnight at 4 °C, followed by secondary antibody staining with Alexa Fluor-568 goat anti-mouse IgG (H+L) (1:1000) (Life Technologies A-11031) for 2 h at room temperature. Coverslips were then washed and mounted using ProLong Gold Antifade reagent containing 4′,6-diamidino-2-phenylindole (DAPI) (Molecular Probes, Invitrogen) for DNA staining. The number of 5-HT^+^ cells per 10 crypts was counted in four different areas for each section. If the section is in poor condition (<25% damaged), one of the four sections is counted on the opposite side. If >25% of the section is damaged, the section is not counted. For colonic organoids-derived monolayer staining, monolayers grown on coverslips in 6-well plates were fixed with 4% PFA, followed by blocking with 2% BSA in PBS, 0.01% Triton X-100 and 0.05% Tween 20 for 1 h at room temperature. Monolayers were stained with anti-rabbit ZO-1 antibody (1:100; Cat. # 40-2200, Thermo Fisher Scientific, Mississauga, Canada), followed by secondary antibody staining with Alexa Fluor-488 donkey anti-rabbit IgG (H+L) (1:1000) (Life Technologies A-21206) for 2 h at room temperature. Coverslips were then washed and mounted using ProLong Gold Antifade reagent containing DAPI (Molecular Probes, Invitrogen) for DNA staining. Images were captured using a Nikon Eclipse 80i microscope and NIS-Elements Basic Research imaging software. Investigators were blinded to the study groups.

### Liquid chromatography with tandem mass spectrometry (LC-MS/MS) sample preparation and method

A 100 µL of serum sample was spiked with 10 µL of 10 μg mL^−1^ Trypan Blue (internal standard). Proteins were precipitated by adding 380 µL of acetonitrile, vortexing for 10 min and centrifuging for 10 min at 16,000 × g. A 450 µL of the supernatant was removed and dried under nitrogen. Samples were reconstituted in 100 µL of water and vortexed for 4 min and centrifuged for 5 min at 16,000 × g. A 75 µL of the supernatant was added to an LC vial with an inset. A 5 µL of sample was injected onto a Phenomenex Kinetex C18 3 × 50 mm column, and separated with the following gradient: A = 10 mmol L^−1^ ammonium acetate, B = 10 mmol L^−1^ ammonium acetate in methanol, 0-min, 85% A, 10 mi, 0% A, 12 min, 0% A, 12.1 min, 85% A, 16 min, 85% A. The flow rate was 400 µL min^−1^. Leftovers from sampling were combined to produce a pooled sample, which was spiked for recovery. The calculated recovery was 90%.

### Bioinformatics and 16s rRNA sequencing

Cecal contents were stored at −80 °C. DNA was extracted and the variable 3 (v3) and v4 gene region of the 16S rRNA gene was amplified as previously described with minor modifications^66,67^. Samples were thawed to room temperature. Approximately 0.2–0.3 g of sample was added to 800 μL of 200 mmol L^−1^ NaPO_4_ (pH 8), and 100 μL of guanidine thiocyanate-ethylenediaminetetraacetic acid (EDTA)–Sarkosyl along with 0.2 g of 0.1 mm glass beads (Mo-Bio, Carlsbad, CA). Mechanical lysis was carried out in a Powerlyzer (Mo-Bio) at 3000 RPMs for 3 min. Enzymatic lysis was performed using 50 μL lysozyme (100 mg mL^−1^), 10 μL RNase A (10 mg mL^−1^), and incubation at 37 °C for 90 min. Next, 25 μL 25% sodium dodecyl sulfate (SDS), 25 μL proteinase K, and 62.5 μL 5 mol L^−1^ NaCl were added to the sample and incubated at 65 °C for 90 min. Following, samples were pelleted at 13,500 × g for 5 min by centrifugation and the supernatant was recovered. Genomic DNA was purified using a MagMAX (Thermo Fisher Scientific) as per the manufacturer’s instructions. The 16S rRNA v3 and v4 gene region was amplified in triplicate using 341F and 806R 16S rRNA primers modified for the Illumina platform with adaptors containing 6-base pair unique barcodes to the reverse primer. Each sample reaction mixture contained 5 pmol L^−1^ of each primer, 200 μmol L^−1^ of each deoxynucleoside triphosphate (dNTP), 1.5 mmol L^−1^ MgCl_2_, and 1U *Taq* polymerase (Life Technologies, Carlsbad). The PCR protocol consisted of an initial denaturation step at 95 °C for 5 min, 30 cycles, each step for 30 s, of 95 °C, 50 °C, and 72 °C, and a final extension step at 72 °C for 7 min. Amplification of the v3 and v4 gene regions was verified by electrophoresis on a 2% agarose gel. Amplicons were then sequenced (2 × 300 paired end) using the Illumina MiSeq platform. Resulting paired-end sequences from Illumina sequencing were processed using DADA2 pipeline^68^. Cutadapt was used to filter and trim adapter sequences and PCR primers from the raw reads with a minimum quality score of 30 and a minimum read length of 100 bp^69^. Sequence variants were then resolved from the trimmed raw reads using DADA2, an accurate sample inference pipeline from 16s amplicon data^68^. DNA sequence reads were filtered and trimmed based on the quality of the reads for each Illumina run separately, error rates were learned, and sequence variants were determined by DADA2. Sequence variant tables were merged to combine all information from separate Illumina runs. Bimeras were removed and taxonomy was assigned using the SILVA database version 1.2.8. Analysis was conducted using the phyloseq^70^ on R and using GraphPad Prism version 5.0 (GraphPad Software, La Jolla, CA). The significance was based on the phylum and genus level, not ASV. The table was filtered to remove taxa whose mean abundance was 10 or less, samples with sampling depth <2500, and those reads not assigned to bacteria or archaea. After filtering, the minimal number of reads per sample was 29,926, and the samples were rarified to 20,000 reads.

### Statistical analysis

All data are expressed as mean or mean ± SD or SEM. Where appropriate, statistical differences between mean values were determined by two-tailed unpaired Student’s *t*-test, one-way ANOVA, or two-way ANOVA with the post hoc Bonferroni’s, Dunnett’s multiple comparison test, or two-tailed Mann–Whitney *U* test using GraphPad Prism version 5.0 (GraphPad Software, La Jolla, CA). *P* < 0.05 was considered statistically significant.

### Reporting summary

Further information on research design is available in the Nature Portfolio Reporting Summary linked to this article.

## Supplementary information

Supplementary Information

Reporting Summary

## Data Availability

All data supporting the findings of this study are provided within the Supplementary Information file and that data are available from the corresponding author. The 16S rRNA sequencing data are available in Sequence Read Archive with BioProject PRJNA896988. Source Data are provided with this paper.

## Source data

Source Data
