# Author Correction: Chronic exposure to synthetic food colorant Allura Red AC promotes susceptibility to experimental colitis via intestinal serotonin in mice

**DOI:** 10.1038/s41467-023-38903-w

**Published:** 2023-05-30

**Authors:** Yun Han Kwon, Suhrid Banskota, Huaqing Wang, Laura Rossi, Jensine A. Grondin, Saad A. Syed, Yeganeh Yousefi, Jonathan D. Schertzer, Katherine M. Morrison, Michael G. Wade, Alison C. Holloway, Michael G. Surette, Gregory R. Steinberg, Waliul I. Khan

**Affiliations:** 1 Department of Pathology and Molecular Medicine, McMaster University, Hamilton, ON Canada; 2 Farncombe Family Digestive Health Research Institute, McMaster University, Hamilton, ON Canada; 3 Department of Biochemistry and Biomedical Sciences, McMaster University, Hamilton, ON Canada; 4 Department of Medicine, McMaster University, Hamilton, ON Canada; 5 Center for Metabolism, Obesity, and Diabetes Research, McMaster University, Hamilton, ON Canada; 6 Department of Pediatrics, McMaster University, Hamilton, ON Canada; 7 Environmental Health, Science and Research Bureau, Health Canada, Ottawa, ON Canada; 8 Department of Obstetrics and Gynecology, McMaster University, Hamilton, ON Canada

**Keywords:** Inflammatory bowel disease, Microbiome

Correction to: *Nature Communications* 10.1038/s41467-022-35309-y, published online 20 December 2022

In this article the word ‘not’ was inadvertently omitted from the sentence “In addition, colonic interleukin (IL)−1β, IL-6, and tumor necrosis factor (TNF)-α, were higher in AR-DSS (Fig. 1l) than their DSS counterparts, while the genes that regulate intestinal epithelial barrier function (zonula occludin-1 [ZO-1; *Tjp1*], and occludin [*Ocln*]), were reduced in AR-DSS compared to their DSS counterparts (Fig. 1m).” in the Results section. The corrected sentence reads “In addition, colonic interleukin (IL)−1β, IL-6, and tumor necrosis factor (TNF)-α, were higher in AR-DSS (Fig. 1l) than their DSS counterparts, while the genes that regulate intestinal epithelial barrier function (zonula occludin-1 [ZO-1; *Tjp1*], and occludin [*Ocln*]), were not reduced in AR-DSS compared to their DSS counterparts (Fig. 1m).”

In addition, the citation to reference 59 in the sentence ”This finding is consistent with previous study that found CS did not induce colitis in mice^59^“ in the Discussion section should instead cite reference 60. The corrected sentence reads “This finding is consistent with previous study that found CS did not induce colitis in mice^60^”.

These errors have been corrected in the HTML and PDF versions of the Article.

