# Supplementary material for: Chronic exposure to synthetic food colorant Allura Red AC promotes susceptibility to experimental colitis via intestinal serotonin in mice
Source: Nat Commun. 2022 Dec 20;13:7617. doi: 10.1038/s41467-022-35309-y (PMC9768151; doi:10.1038/s41467-022-35309-y)

Supplementary Information

Chronic exposure to synthetic food colorant Allura Red AC promotes susceptibility to experimental colitis via intestinal serotonin in mice

Yun Han Kwon[1,2], Suhrid Banskota[1,2], Huaqing Wang[1,2], Laura Rossi[2,3], Jensine A. Grondin[1,2], Saad A. Syed[2,3,4], Yeganeh Yousefi[1,2], Jonathan D. Schertzer[3,5], Katherine M. Morrison[5,6], Michael G. Wade[7], Alison C. Holloway[5,8], Michael G. Surette[2,3,4], Gregory R. Steinberg[3,4,5], & Waliul I. Khan[1,2,*]

[1]Department of Pathology and Molecular Medicine, McMaster University; Hamilton, Ontario, Canada.

[2]Farncombe Family Digestive Health Research Institute, McMaster University; Hamilton, Ontario, Canada.

[3]Department of Biochemistry and Biomedical Sciences, McMaster University; Hamilton, Ontario, Canada.

[4]Department of Medicine, McMaster University; Hamilton, Ontario, Canada

[5]Center for Metabolism, Obesity, and Diabetes Research, McMaster University; Hamilton, Ontario, Canada.

[6]Department of Pediatrics, McMaster University; Hamilton, Ontario, Canada.

[7]Environmental Health, Science and Research Bureau, Health Canada; Ottawa, Ontario, Canada

[8]Department of Obstetrics and Gynecology, McMaster University; Hamilton, Canada.

***Correspondence**: Waliul I. Khan, MBBS, PhD, FRCPath

E-mail: khanwal@mcmaster.ca

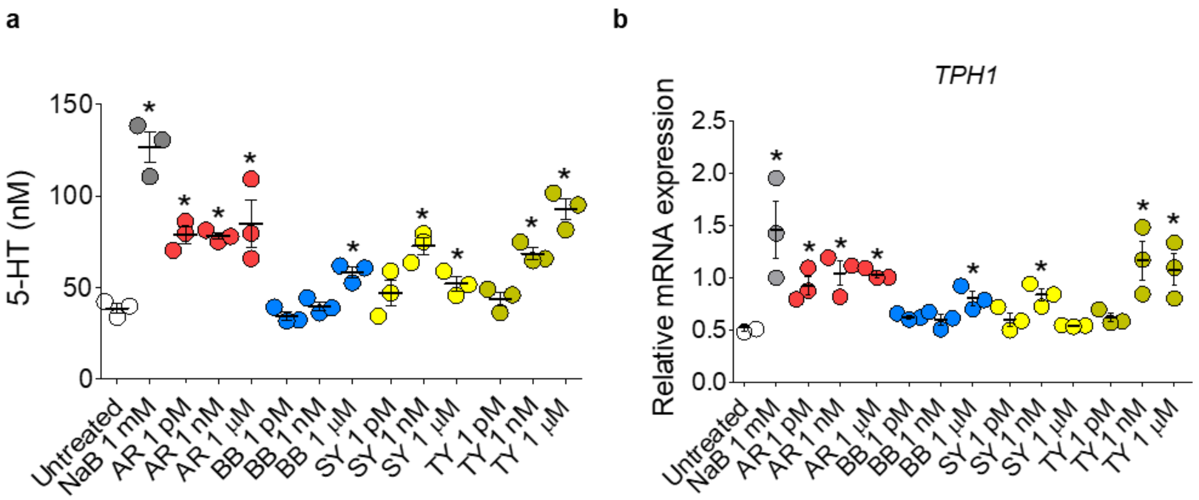

Supplementary Figure 1. Synthetic colorants promote 5-HT secretion and induce *TPH1* mRNA expression in BON cells. BON cells were treated for 24 hours with synthetic colorants at three different concentrations (1 pmol L^{-1}, 1 nmol L^{-1}, and 1 µmol L^{-1}). Sodium butyrate (NaB; 1 mmol L^{-1}) was used as a positive control. **a** 5-HT levels in the culture supernatant, and **b** *TPH1* mRNA levels. Data were analyzed by one-way ANOVA with *post hoc* Dunnett's test and are expressed as mean ± SEM of 3 independent experiments (n = 3). Significance denoted by *p < 0.05, where *p < 0.05 versus Untreated. Source data are provided as a Source Data File.

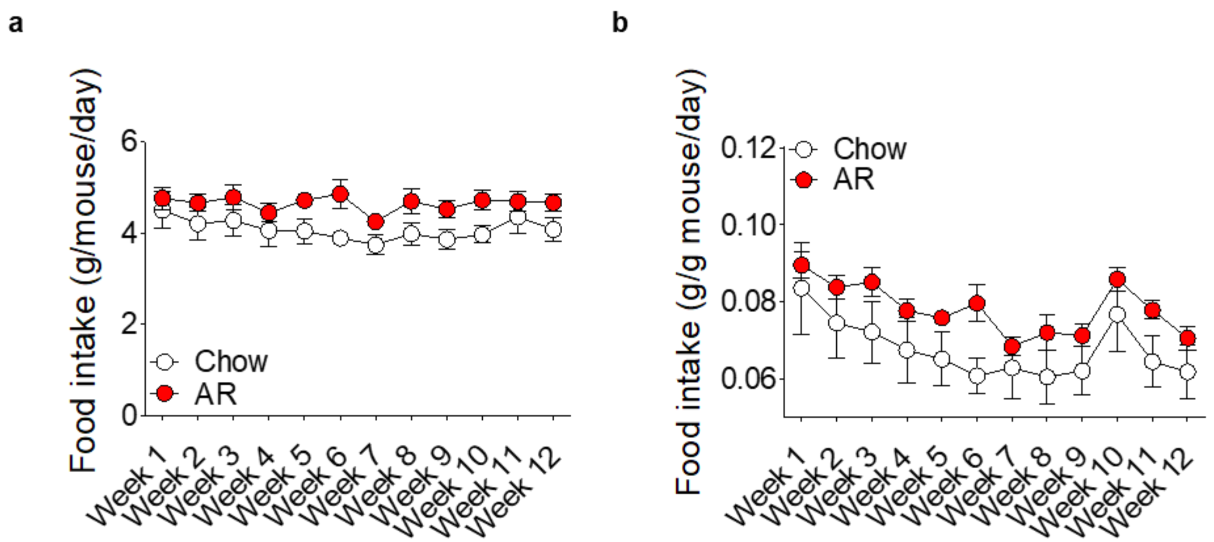

Supplementary Figure 2. Food intake measurement in C57BL/6 mice during AR exposure via normal chow diet for 12 weeks. Diet was freshly replaced every week and consumption rate was measured. **a** Food intake represented in g per mouse per day. **b** Food intake is represented in g per g of mouse per day. Data are expressed as mean ± SEM (n = 9 for Chow; n = 13 for AR). Source data are provided as a Source Data File.

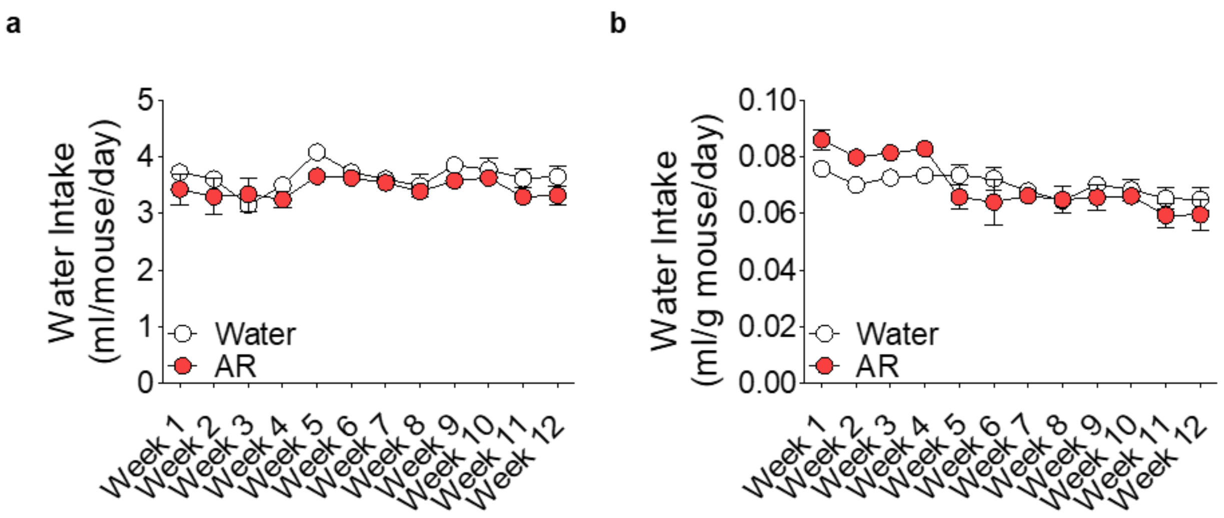

Supplementary Figure 3. Water intake measurement in C57BL/6 mice during AR exposure via normal drinking water for 12 weeks. Drinking water was freshly replaced every week and consumption rate was measured. **a** Water intake represented in ml per mouse per day. **b** Water intake is represented in ml per g of mouse per day. Data are expressed as mean ± SEM (n = 10 for Water; n = 14 for AR). Source data are provided as a Source Data File.

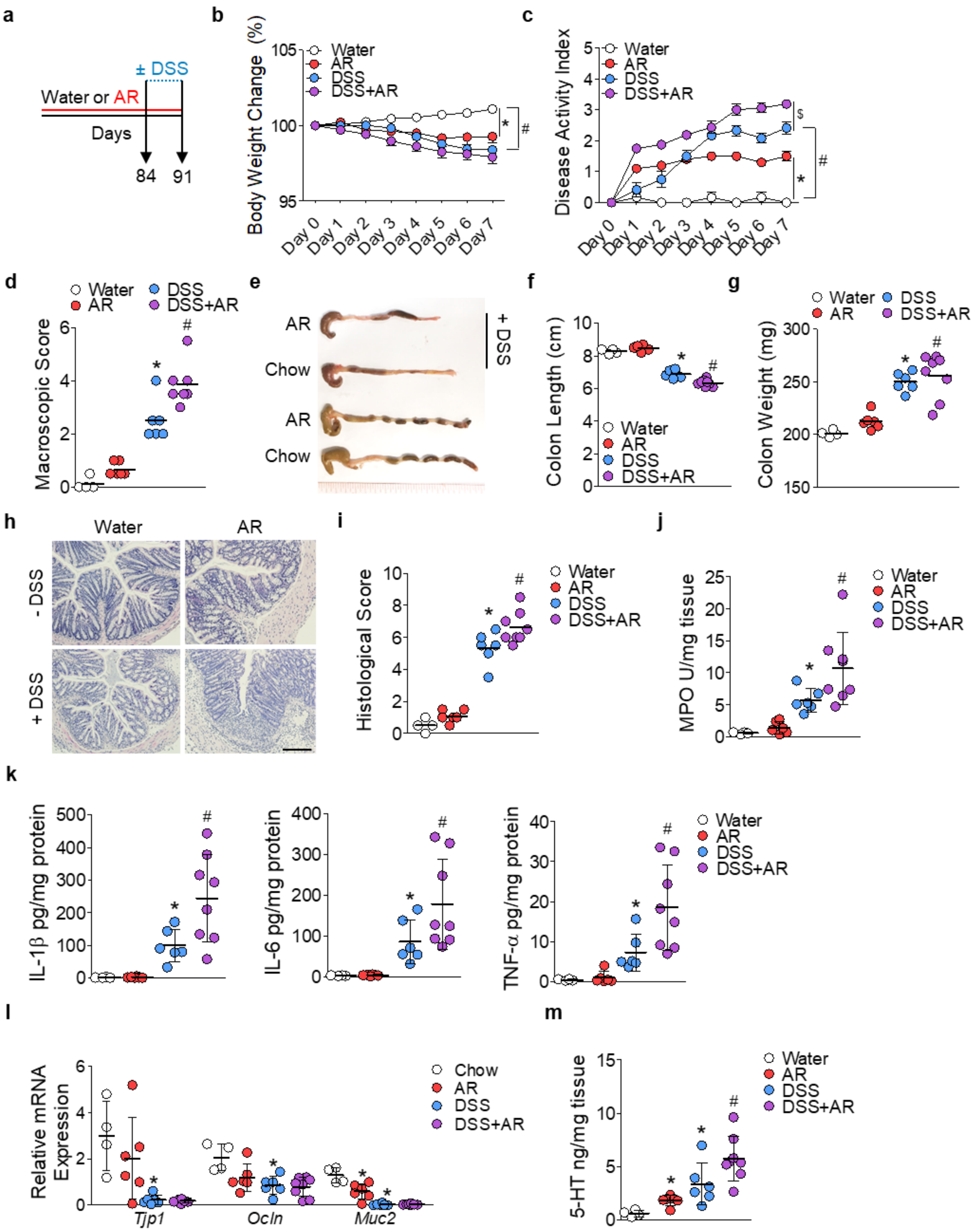

a
± DSS
Water or AR
Days
84
91
b
Body Weight Change (%)
Water
AR
DSS
DSS+AR
c
Disease Activity Index
d
Macroscopic Score
e
AR
Chow
+ DSS
f
Colon Length (cm)
g
Colon Weight (mg)
h
Water
AR
- DSS
+ DSS
i
Histological Score
j
MPO U/mg tissue
k
IL-1β pg/mg protein
IL-6 pg/mg protein
TNF-α pg/mg protein
l
Relative mRNA Expression
Chow
Tjp1
Ocln
Muc2
m
5-HT ng/mg tissue

Supplementary Figure 4. AR exposure via normal drinking water exacerbates the development of DSS-induced colitis in C57BL/6 mice. C57BL/6 mice were either received normal drinking water or exposed to 100 ppm AR via water for 12 weeks (84 days) prior to induction of acute colitis by 3.5% DSS for 7 days. During DSS, mice were continuously exposed to AR. **a** Schematic illustration of the experimental design. **b** Body weight change during DSS. **c** Disease activity index (DAI) during DSS. **d** Macroscopic score. **e** A representative image of colons. **f** Colon length (cm). **g** Colon weight (mg). **h** Representative images of H&E-stained colon sections on day 7 post-DSS; scale bar: 100 μm. **i** Histological score. **j** Colonic MPO levels. **k** Colonic IL-1β, IL-6, and TNF-α levels. **l** Relative mRNA expression of intestinal epithelial barrier function related genes. **m** Colonic 5-HT level. **b** and **c** Data were analyzed by two-way ANOVA with *post hoc* Bonferroni's test and are expressed as mean ± SEM (n = 4 for Water; n = 6 for AR, and DSS; n = 8 for DSS+AR). **d**–**m** Data were analyzed by one-way ANOVA with *post hoc* Bonferroni's test and are expressed as mean or mean ± SD (n = 4 for Water; n = 6 for AR, and DSS; n = 8 for DSS+AR). Significance denoted by $^{*}p < 0.05$, $^{\#}p < 0.05$, where $^{*}p < 0.05$ versus Water, and $^{\#}p < 0.05$ versus DSS. Source data are provided as a Source Data File.

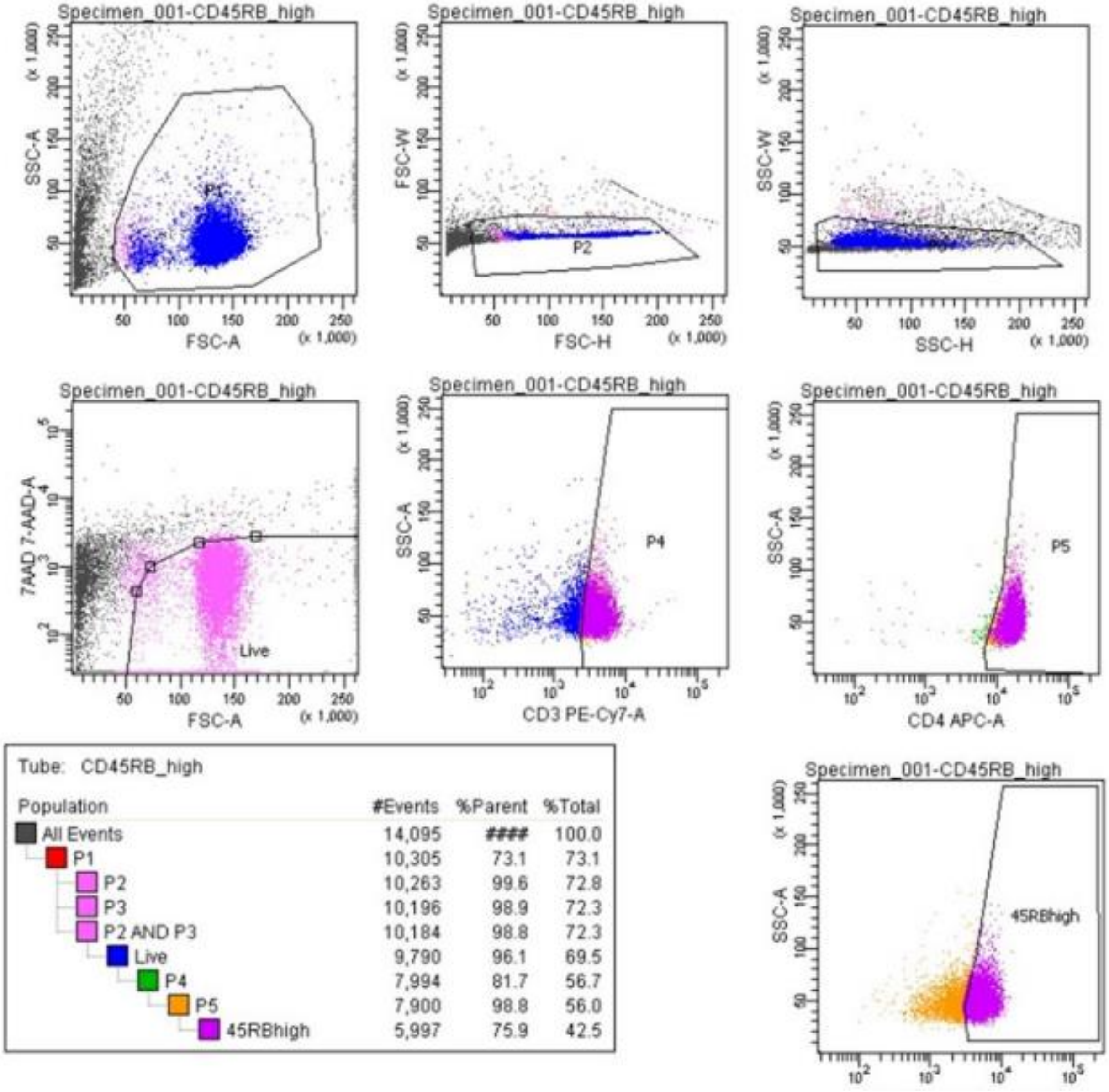

Population	#Events	%Parent	%Total
All Events	14,095	####	100.0
P1	10,305	73.1	73.1
P2	10,263	99.6	72.8
P3	10,196	98.9	72.3
P2 AND P3	10,184	98.8	72.3
Live	9,790	96.1	69.5
P4	7,994	81.7	56.7
P5	7,900	98.8	56.0
45RBhigh	5,997	75.9	42.5

Supplementary Figure 5. Sorting strategy of CD4$^+$CD45RB^{hi} T cells. Naïve CD4$^+$ T cells were stained with antibody cocktail containing anti-CD3-PE-Cy7, anti-CD4-APC, and anti-CD45RB-FITC antibodies, and CD4$^+$CD45RB^{hi} cells were gated as above.

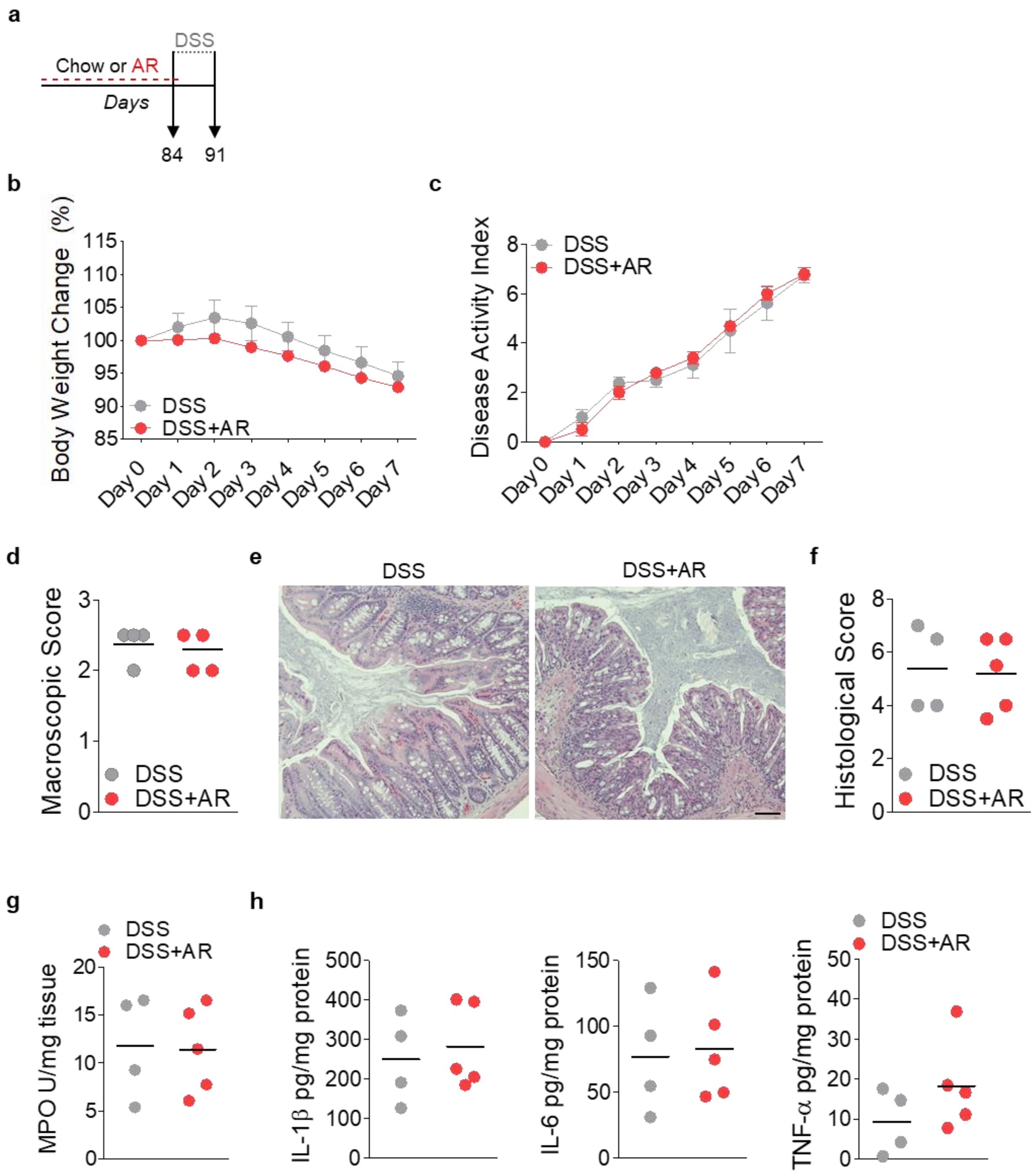
a
DSS
Chow or AR
Days
84
91
b
Body Weight Change (%)
115
110
105
100
95
90
85
DSS
DSS+AR
Day 0
Day 1
Day 2
Day 3
Day 4
Day 5
Day 6
Day 7
c
Disease Activity Index
8
6
4
2
0
DSS
DSS+AR
d
Macroscopic Score
3
2
1
0
DSS
DSS+AR
e
DSS
DSS+AR
f
Histological Score
8
6
4
2
0
DSS
DSS+AR
g
MPO U/mg tissue
20
15
10
5
0
DSS
DSS+AR
h
IL-1β pg/mg protein
500
400
300
200
100
0
IL-6 pg/mg protein
150
100
50
0
TNF-α pg/mg protein
50
40
30
20
10
0
DSS
DSS+AR

Supplementary Figure 6. Intermittent exposure of AR does not enhance susceptibility to DSS-induced colitis in C57BL/6 mice. C57BL/6 mice were either fed normal chow diet or intermittently exposed to 100 ppm AR via diet for one day per week for 12 weeks (84 days) prior to induction of acute colitis with 3.5% DSS for 7 days. Mice were only exposed to AR on the first day of DSS. **a** Schematic illustration of the experimental design. **b** Body weight changes during DSS. **c** Disease activity index (DAI) during DSS. **d** Macroscopic score. **e** Representative images of H&E-stained colon sections on day 7 post-DSS; scale bar: 100 μm. **f** Histological score. **g** Colonic MPO levels. **h** Colonic IL-1β, IL-6, and TNF-α levels. **b** and **c** Data were analyzed by two-way ANOVA with *post hoc* Bonferroni's test and are expressed as mean ± SEM (n = 4 for DSS; n = 5 for DSS+AR). **d**–**h** Data were analyzed by two-tailed unpaired Student's *t*-test and are expressed as mean or mean ± SD (n = 4 for DSS; n = 5 for DSS+AR). Source data are provided as a Source Data File.

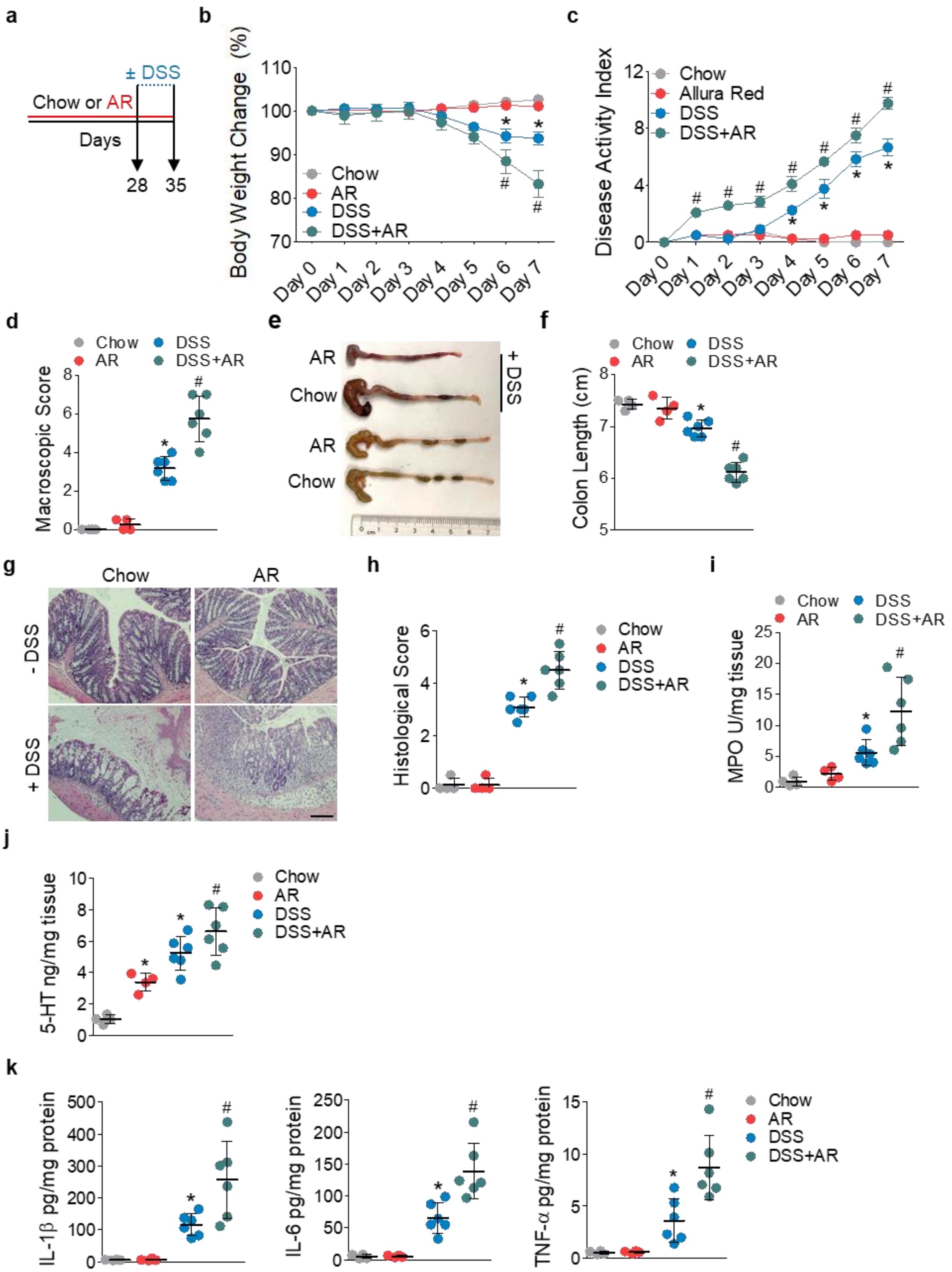
a
± DSS
Chow or AR
Days
28
35
b
Body Weight Change (%)
Chow
AR
DSS
DSS+AR
Day 0
Day 1
Day 2
Day 3
Day 4
Day 5
Day 6
Day 7
c
Disease Activity Index
Chow
Allura Red
DSS
DSS+AR
d
Macroscopic Score
e
AR
Chow
+ DSS
f
Colon Length (cm)
g
Chow
AR
- DSS
+ DSS
h
Histological Score
i
MPO U/mg tissue
j
5-HT ng/mg tissue
k
IL-1β pg/mg protein
IL-6 pg/mg protein
TNF-α pg/mg protein

Supplementary Figure 7. AR exposure during early life increases the susceptibility to DSS-induced colitis in later life. Four-week-old C57BL/6 mice were either fed normal chow diet or exposed to 100 ppm AR via diet for 4 weeks (28 days) prior to induction of acute colitis by 3.5% DSS for 7 days. During DSS, mice were not exposed to AR, but fed normal chow diet. **a** Schematic illustration of the experimental design. **b** Body weight changes during DSS. **c** Disease activity index (DAI) during DSS. **d** Macroscopic score. **e** A representative image of colons. **f** Colonic length (cm). **g** Representative images of H&E-stained colon sections on day 7 post-DSS; scale bar: 100 µm. **h** Histological score. **i** Colonic MPO levels. **j** Colonic 5-HT levels. **k** Colonic IL-1β, IL-6, and TNF-α levels. **b** and **c** Data were analyzed by two-way ANOVA with *post hoc* Bonferroni's test and are expressed as mean ± SEM (n = 4 for Chow, and AR; n = 6 for DSS, and DSS+AR). **d**–**k** Data were analyzed by one-way ANOVA with *post hoc* Bonferroni's test and are expressed as mean or mean ± SD (n = 4 for Chow, and AR; n = 6 for DSS, and DSS+AR). Significance denoted by $^{*}p < 0.05$, $^{\#}p < 0.05$, where $^{*}p < 0.05$ versus Chow, and $^{\#}P < 0.05$ versus DSS. Source data are provided as a Source Data File.

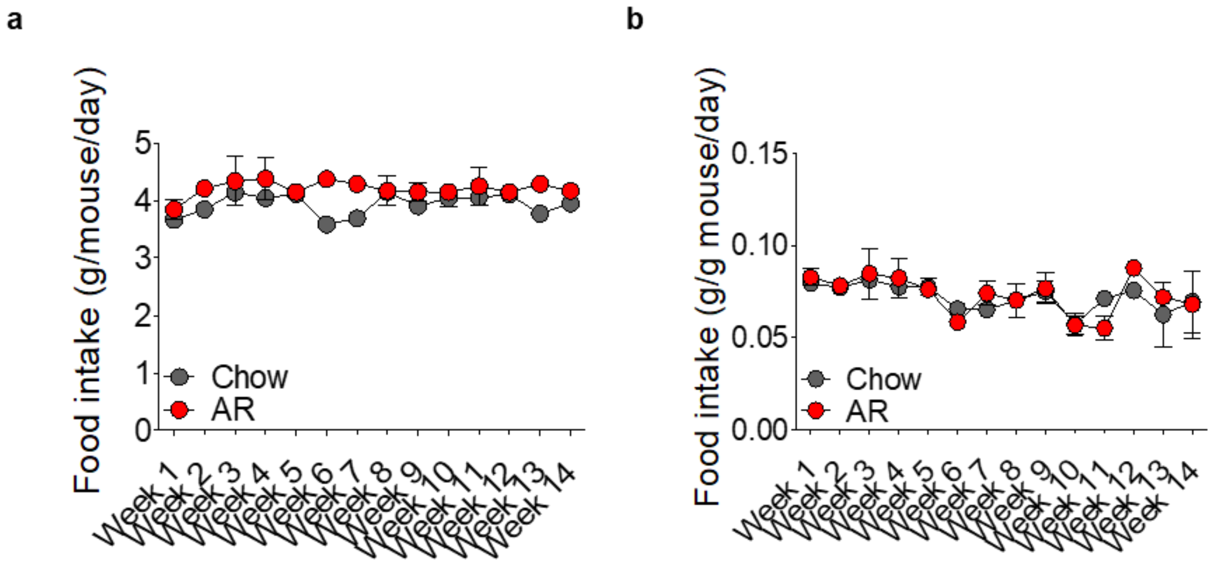

Supplementary Figure 8. Food intake measurement in naïve C57BL/6 mice exposed to AR via normal chow diet for 14 weeks. Diet was freshly replaced every week and consumption rate was measured. **a** Food intake represented in g per mouse per day. **b** Food intake is represented in g per g of mouse per day. Data are expressed as mean ± SEM (n = 4 for Chow; n = 5 for AR). Source data are provided as a Source Data File.

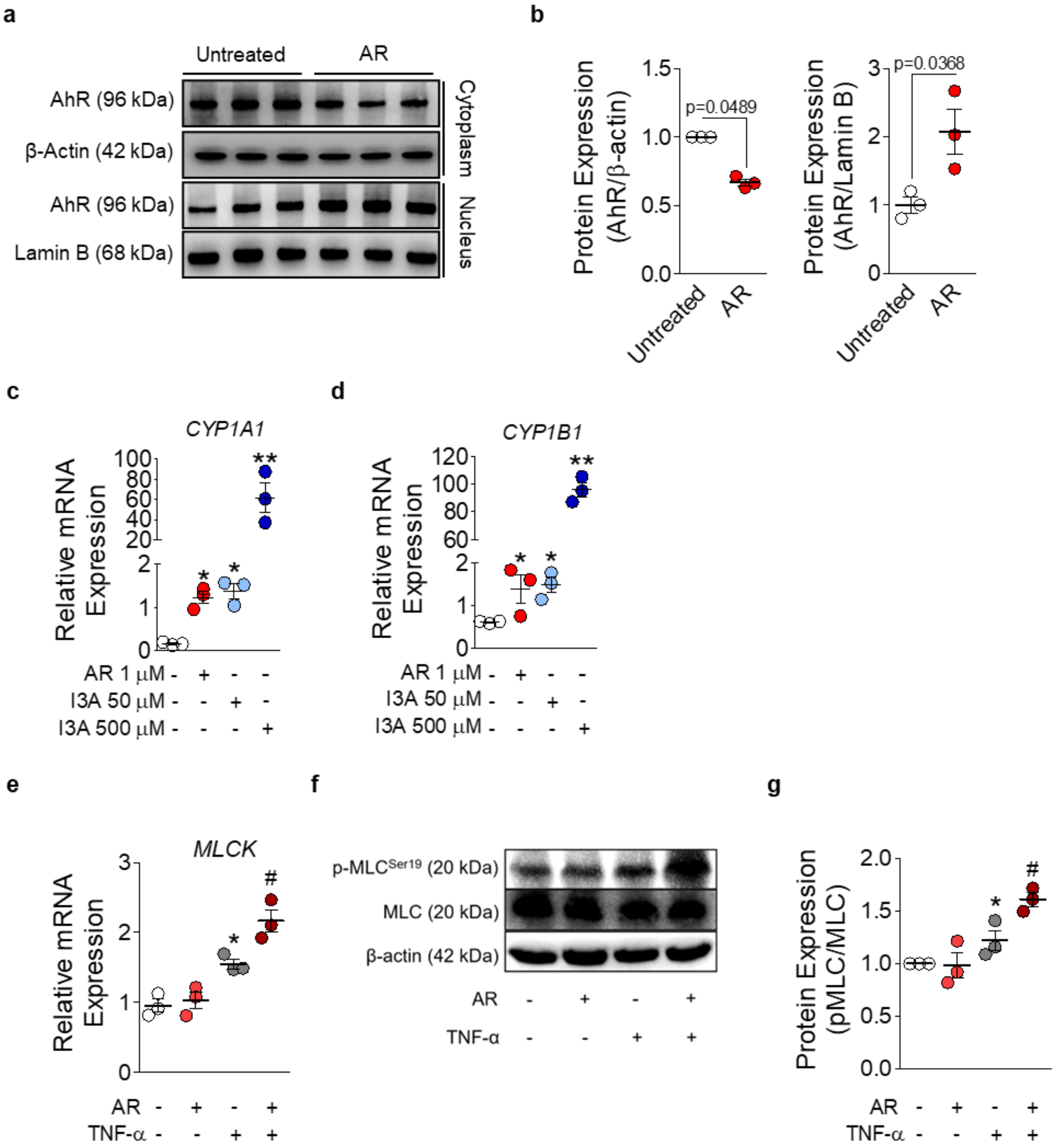
a
Untreated
AR
AhR (96 kDa)
β-Actin (42 kDa)
AhR (96 kDa)
Lamin B (68 kDa)
Cytoplasm
Nucleus
b
Protein Expression (AhR/β-actin)
p=0.0489
Untreated
AR
Protein Expression (AhR/Lamin B)
p=0.0368
Untreated
AR
c
CYP1A1
Relative mRNA Expression
AR 1 μM - + - -
I3A 50 μM - - + -
I3A 500 μM - - - +
d
CYP1B1
Relative mRNA Expression
AR 1 μM - + - -
I3A 50 μM - - + -
I3A 500 μM - - - +
e
MLCK
Relative mRNA Expression
AR - + - +
TNF-α - - + +
f
p-MLCSer19 (20 kDa)
MLC (20 kDa)
β-actin (42 kDa)
AR - + - +
TNF-α - - + +
g
Protein Expression (pMLC/MLC)
AR - + - +
TNF-α - - + +

Supplementary Figure 9. AR up-regulates *CYP1A1*/*CYP1B1* mRNA expression and activates MLCK pathway in HT-29 cells. HT-29 cells were treated for 24 hours with AR (1 µmol L^{-1}). Indole-3-carboxaldehyde (I3A) was used as a positive control. In another experiment, HT-29 cells were pre-treated for 1 hour with TNF-α (10 ng mL^{-1}), followed by 24 hours of AR treatment. **a** Representative western blot analysis of nuclear translocation of AhR. β-actin was used for quantifying cytoplasmic proteins. Lamin B was used for quantifying nuclear proteins. Uncropped blots are provided in the Supplementary Figure 17. **b** Relative density of nuclear and cytoplasmic AhR. **c** *CYP1A1* mRNA levels. **d** *CYP1B1* mRNA levels. **e** *MLCK* mRNA levels. **f** Representative western blot analysis of $pMLC^{Ser19}$, MLC and β-actin. Uncropped blots are provided in the Supplementary Figure 17. **g** Relative density of $pMLC^{Ser19}$/MLC. **b** Data were analyzed by two-tailed unpaired Student's *t*-test and are expressed as mean ± SEM of 3 independent experiments (n = 3). **c**–**d** Data were analyzed by one-way ANOVA with *post hoc* Dunnett's test and are expressed as mean ± SEM of 3 independent experiments (n = 3) **e**–**g** Data were analyzed by one-way ANOVA with *post hoc* Bonferroni's test and are expressed as mean ± SEM of 3 independent experiments (n = 3). Significance denoted by $^{*}p < 0.05$, $^{\#}p < 0.05$ unless otherwise provided. $^{*}p < 0.05$ versus untreated, and $^{\#}p < 0.05$ versus TNF-α. Source data are provided as a Source Data File.

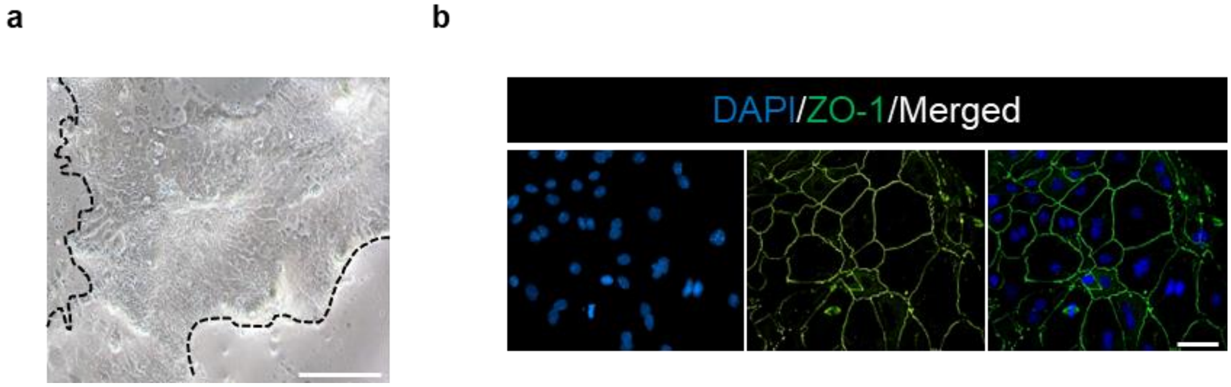
a
b
DAPI/ZO-1/Merged

Supplementary Figure 10. A functional 2D monolayer derived from murine colonic organoids. a A representative brightfield image of 2D monolayer derived from murine colonic organoids. **b** Representative immunofluorescence images of a functional 2D monolayer stained for ZO-1 (green) and DAPI (blue). Scale bar: 50 μm. Data are representative of 2 independent experiments (n = 2).

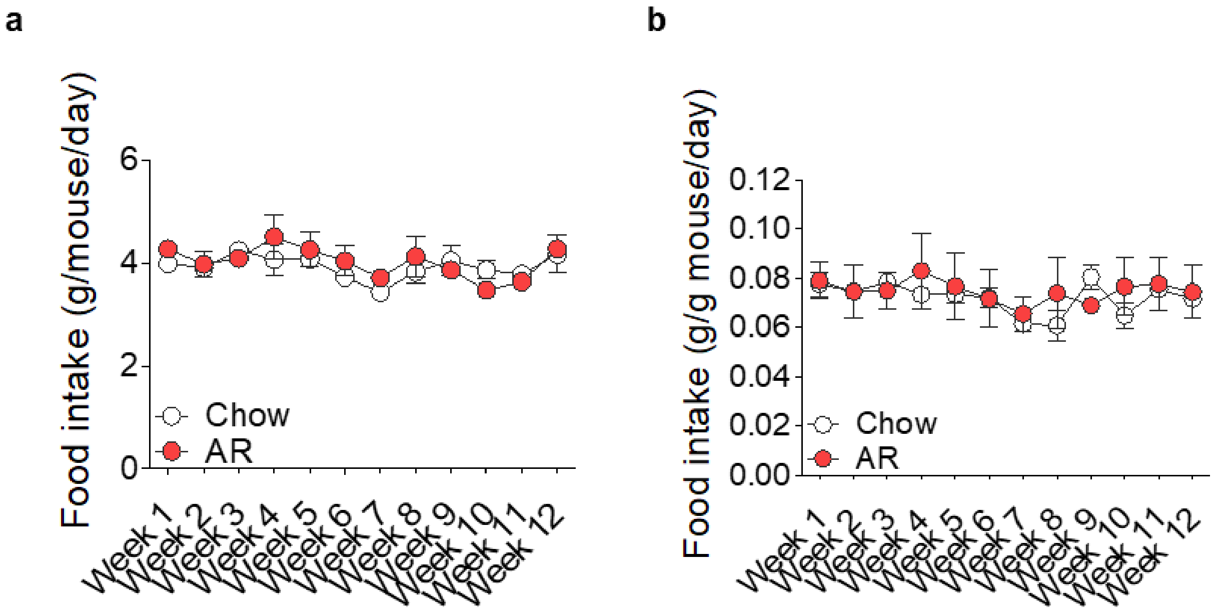

Supplementary Figure 11. Food intake measurement in Tph1-deficient mice exposed to AR via normal chow diet for 12 weeks. Diet was freshly replaced every week and consumption rate was measured. **a** Food intake represented in g per mouse per day. **b** Food intake is represented in g per g of mouse per day. Data are expressed as mean ± SEM (n = 8 for Chow; n = 9 for AR). Source data are provided as a Source Data File.

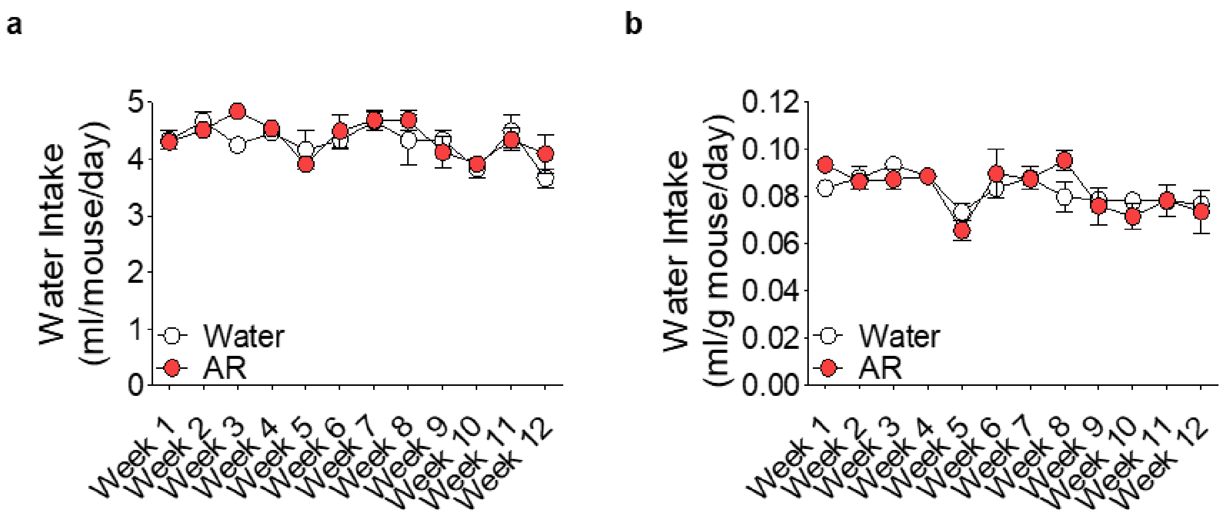

Supplementary Figure 12. Water intake measurement in Tph1-deficient mice exposed to AR via normal drinking water for 12 weeks. Drinking water was freshly replaced every week and consumption rate was measured. **a** Water intake represented in ml per mouse per day. **b** Water intake is represented in ml per g of mouse per day. Data are expressed as mean ± SEM (n = 8 for Chow; n = 8 for AR). Source data are provided as a Source Data File.

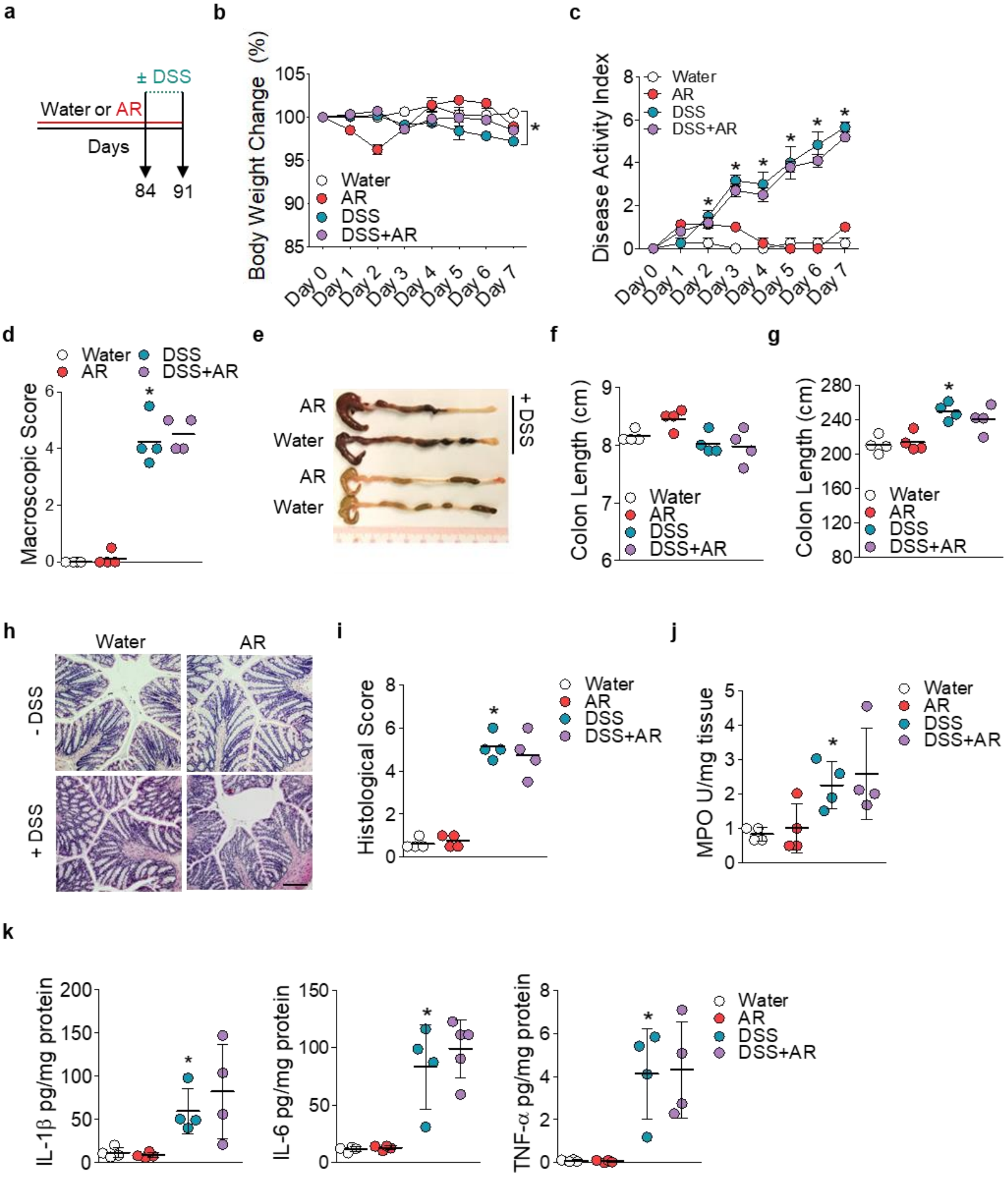
a
± DSS
Water or AR
Days
84
91
b
Body Weight Change (%)
Water
AR
DSS
DSS+AR
Day 0
Day 1
Day 2
Day 3
Day 4
Day 5
Day 6
Day 7
c
Disease Activity Index
d
Macroscopic Score
e
AR
Water
AR
Water
+ DSS
f
Colon Length (cm)
g
Colon Length (cm)
h
Water
AR
- DSS
+ DSS
i
Histological Score
j
MPO U/mg tissue
k
IL-1β pg/mg protein
IL-6 pg/mg protein
TNF-α pg/mg protein

133

Supplementary Figure 13. AR exposure via normal drinking water does not exacerbate DSS-induced colitis in Tph1-deficient mice. *Tph1*$^{-/-}$ mice were either received normal drinking water or exposed to 100 ppm AR via normal drinking water for 12 weeks (84 days) prior to induction of acute colitis by 3.5% DSS for 7 days. During DSS, mice were continuously exposed to AR. **a** Schematic illustration of the experimental design. **b** Body weight change during DSS. **c** Disease activity index (DAI) during DSS. **d** Macroscopic score. **e** A representative image of colons. **f** Colon length (cm). **g** Colon weight (mg). **h** Representative images of H&E-stained colon sections on day 7 post-DSS; scale bar: 100 µm. **i** Histological score. **j** Colonic MPO levels. **k** Colonic IL-1β, IL-6, and TNF-α levels. **b** and **c** Data were analyzed by two-way ANOVA with *post hoc* Bonferroni's test and are expressed as mean ± SEM (n = 4 per group). **d**–**k** Data were analyzed by one-way ANOVA with *post hoc* Bonferroni's test and are expressed as mean or mean ± SD (n = 4 per group). Significance denoted by *p < 0.05, where *p < 0.05 versus Water. Source data are provided as a Source Data File.

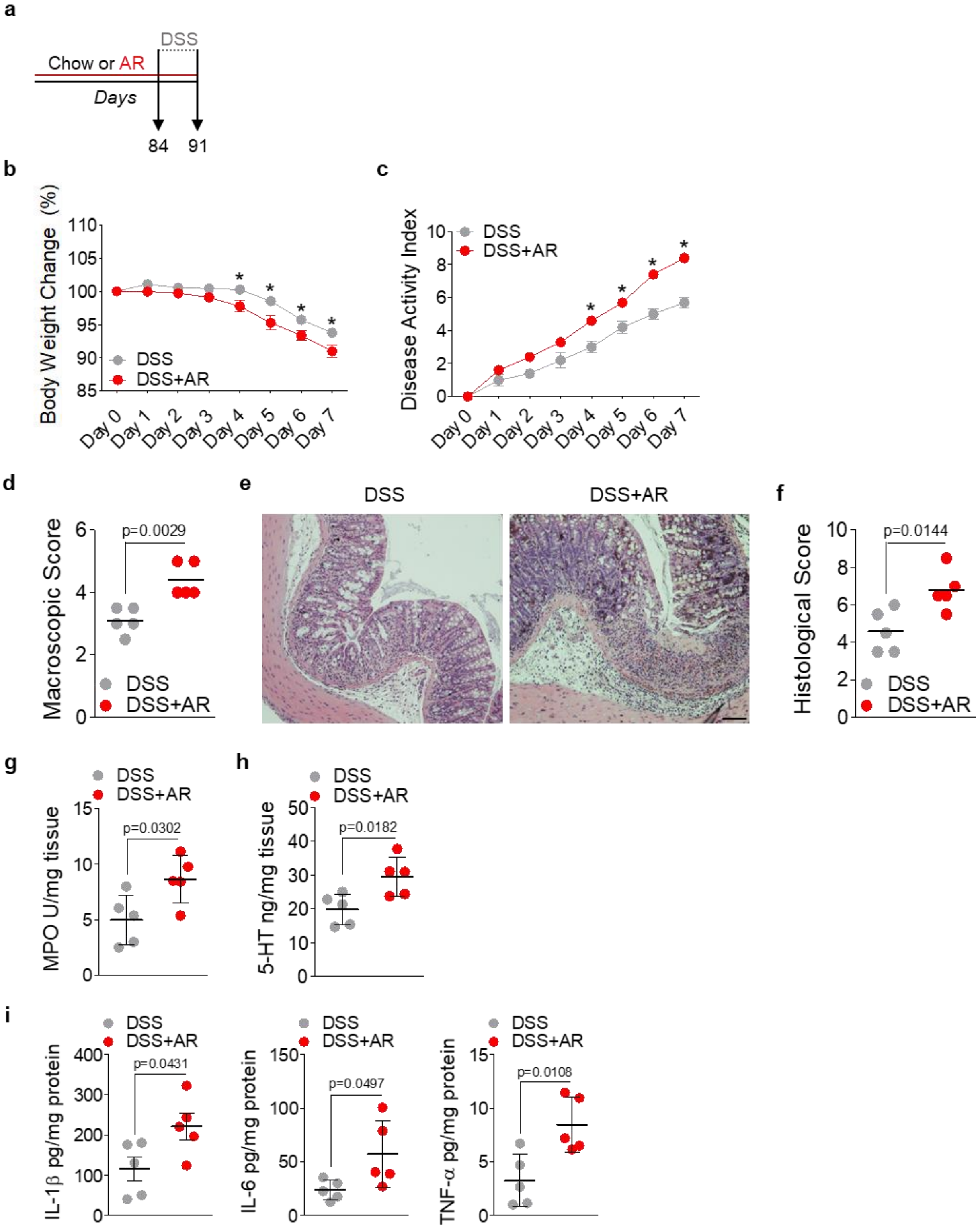
a
DSS
Chow or AR
Days
84
91
b
Body Weight Change (%)
DSS
DSS+AR
c
Disease Activity Index
DSS
DSS+AR
d
Macroscopic Score
p=0.0029
DSS
DSS+AR
e
DSS
DSS+AR
f
Histological Score
p=0.0144
DSS
DSS+AR
g
MPO U/mg tissue
p=0.0302
DSS
DSS+AR
h
5-HT ng/mg tissue
p=0.0182
DSS
DSS+AR
i
IL-1β pg/mg protein
p=0.0431
IL-6 pg/mg protein
p=0.0497
TNF-α pg/mg protein
p=0.0108
DSS
DSS+AR

Supplementary Figure 14. Exposing SERT-deficient mice to AR exacerbates the development of DSS-induced colitis. C57BL/6 mice were either fed normal chow diet or exposed to 100 ppm AR via diet for 12 weeks (84 days) prior to induction of acute colitis by 3.5% DSS for 7 days. During DSS, mice were continuously exposed to AR. **a** Schematic illustration of the experimental design. **b** Body weight change during DSS. **c** Disease activity index (DAI) during DSS. **d** Macroscopic score. **e** Representative images of H&E-stained colon sections on day 7 post-DSS; scale bar: 100 µm. **f** Histological score. **g** Colonic MPO levels. **h** Colonic 5-HT levels. **i** Colonic IL-1β, IL-6, and TNF-α levels. **b** and **c** Data were analyzed by two-way ANOVA with *post hoc* Bonferroni's test and are expressed as mean ± SEM (n = 5 per group). **d**–**i** Data were analyzed by two-tailed unpaired Student's *t*-test and are expressed as mean or mean ± SD (n = 5 per group). Significance denoted by *$p < 0.05$ unless otherwise provided, where *$p < 0.05$ versus DSS. Source data are provided as a Source Data File.

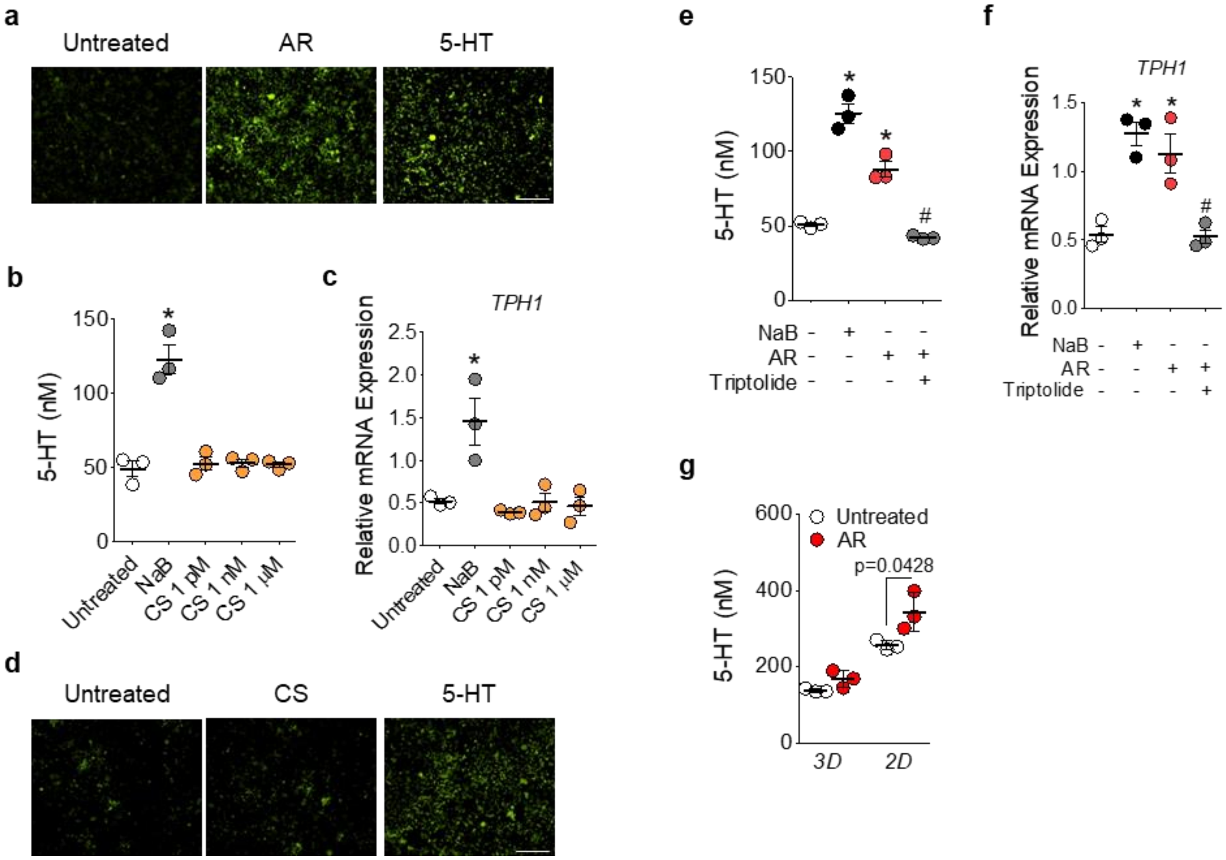
a
Untreated
AR
5-HT
b
5-HT (nM)
150
100
50
0
*
Untreated
NaB
CS 1 pM
CS 1 nM
CS 1 μM
c
TPH1
Relative mRNA Expression
2.5
2.0
1.5
1.0
0.5
0.0
*
Untreated
NaB
CS 1 pM
CS 1 nM
CS 1 μM
d
Untreated
CS
5-HT
e
5-HT (nM)
150
100
50
0
*
*
#
NaB - + - -
AR - - + +
Triptolide - - - +
f
TPH1
Relative mRNA Expression
1.5
1.0
0.5
0.0
*
*
#
NaB - + - -
AR - - + +
Triptolide - - - +
g
5-HT (nM)
600
400
200
0
Untreated
AR
p=0.0428
3D
2D

Supplementary Figure 15. AR, but not CS, induces ROS generation via NF-κB and increases 5-HT secretion in BON cells, and increases 5-HT secretion in murine colonic organoid-derived 2D monolayer. a Representative fluorescence images of intracellular reactive oxygen species (ROS) detected using 2',7'-dichlorofluorescin diacetate (DCF-DA) in BON cells treated for 24 hours with AR (1 µmol L^{-1}) or 5-HT (10 µmol L^{-1}). 5-HT was used as a positive control for ROS induction; scale bar: 50 µm. Data are representative of 2 independent experiments (n = 2). **b** 5-HT levels in the culture supernatant, and **c** *TPH1* mRNA levels, of BON cells treated for 24 hours with three concentrations of *p*-Cresidinesulfonic acid (CS). NaB (1 mmol L^{-1}) was used as positive control for 5-HT secretion. **d** Representative fluorescence images of intracellular ROS detected using DCF-DA in BON cells treated for 24 hours with CS (1 µmol L^{-1}) or 5-HT (10 µmol L^{-1}); scale bar: 50 µm. Data are representative of 2 independent experiments (n = 2). **e** 5-HT level in the culture supernatant, and **f** *TPH1* mRNA levels, of BON cells pre-treated for 1 hour with triptolide (20 nmol L^{-1}), followed by 24 hours of AR treatment (1 µmol L^{-1}). NaB (1 mmol L^{-1}) was used as a positive control. **g** 5-HT levels in mouse colonic organoids and the 2D monolayer. **b** and **c** Data were analyzed by one-way ANOVA with *post hoc* Dunnett's test and are expressed as mean ± SEM of 3 independent experiments (n = 3). **e** and **f** Data were analyzed by one-way ANOVA with *post hoc* Bonferroni's test and are expressed as mean ± SEM of 3 independent experiments (n = 3). **g** Data were analyzed by two-tailed unpaired Student's *t*-test. Data are expressed as mean ± SD and representative of 2 independent experiments (n = 2). Significance denoted by $^*p < 0.05$, $^{\#}p < 0.05$ unless otherwise provided, where $^*p < 0.05$ versus Untreated, and $^{\#}p < 0.05$ versus AR. Source data are provided as a Source Data File.

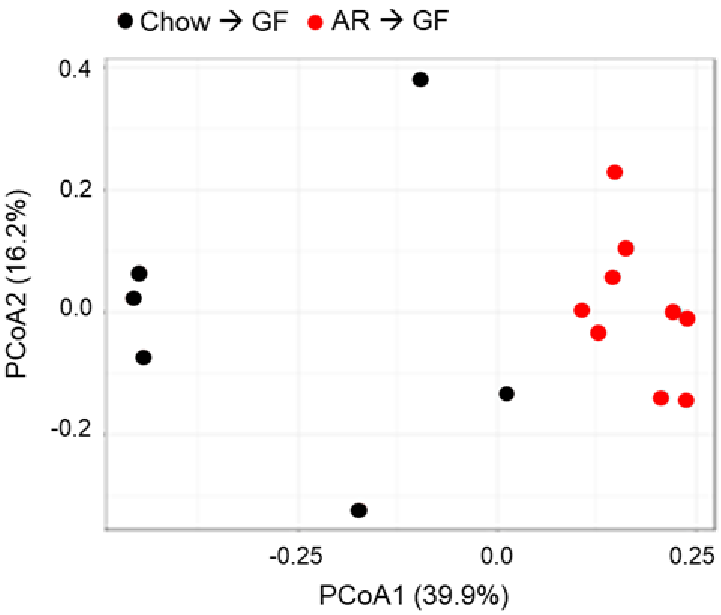

Chow → GF
AR → GF
PCoA2 (16.2%)
PCoA1 (39.9%)
0.4
0.2
0.0
-0.2
-0.25
0.0
0.25

Supplementary Figure 16. Bray-Curtis dissimilarity showing persistence of transplanted microbiota profiles after 21-day colonization in GF mice. 16S rRNA bacterial profiling at the v3-v4 region using feces collected from the recipient GF mice on day 21 was carried out. Bray-Curtis dissimilarity revealed each group of mice (n = 6 for Chow → GF; n = 9 for AR → GF) possessed distinct microbiota as evidenced by two separate clustering pattern. Source data are provided as a Source Data File.

Supplementary Table 1. qRT-PCR Mouse Primers

	Forward (5'-3')	Reverse (5'-3')
18S	GTAACCCGTTGAACCCCATT	CCATCCAATCGGTAGTAGCG
Defb3	GGATCCATTACCTTCTGTTTGC	ATTTGAGGAAAGGAACTCCAC
Il22	TGTCCGGCTCATCGGGGAGA	ACAGCAGGTCCAGTTCCCCA
Mlck	GCGTGATCAGCCTGTTCTTTCTAA	GCCCCATCTGCCCTTCTTTGACC
Muc2	CTGACCAAGAGCGAACACAA	CATGACTGGAAGCAACTGGA
Ocln	ATGTCCGGCCGATGCTCTCTC	CTTTGGCTGCTCTTGGGTCTGTAT
Pparg	CTGCTCAAGTATGGTGTCCATGA	ATGAGGACTCCATCTTTATTCA
Reg3γ	CCGTGCCTATGGCTCCTATTG	GCACAGACACAAGATGTCCTG
Tjp1	ACCCGAAACTGCTGCTGTGGATAG	AAATGGCGGGCAGAACTTGTGTA

Supplementary Table 2. qRT-PCR Human Primers

	Forward (5'-3')	Reverse (5'-3')
18S	TCCACAGGAGGCCTACACGCC	TTTCCGCCGCCCATCGATGTT
CYP1A1	GGTCAAGGAGCACTACAAAACC	TGGACATTGGCGTTCTCAT
CYP1B1	CACTGACATCTTCGGCG	ACCTGATCCAATTCTGCCTG
MLCK	AGGCCAAGGACTTTGTTTCC	TTCAGCCACTCGTGTTTCAG
TPH1	TGCCCTTGCTAAGTTCAGCAGGA	AGCAAGAGATGGCCCAGACCTCC

Supplementary Figure 17. Uncropped blots

Uncropped blots in Supplementary Figure 9a.

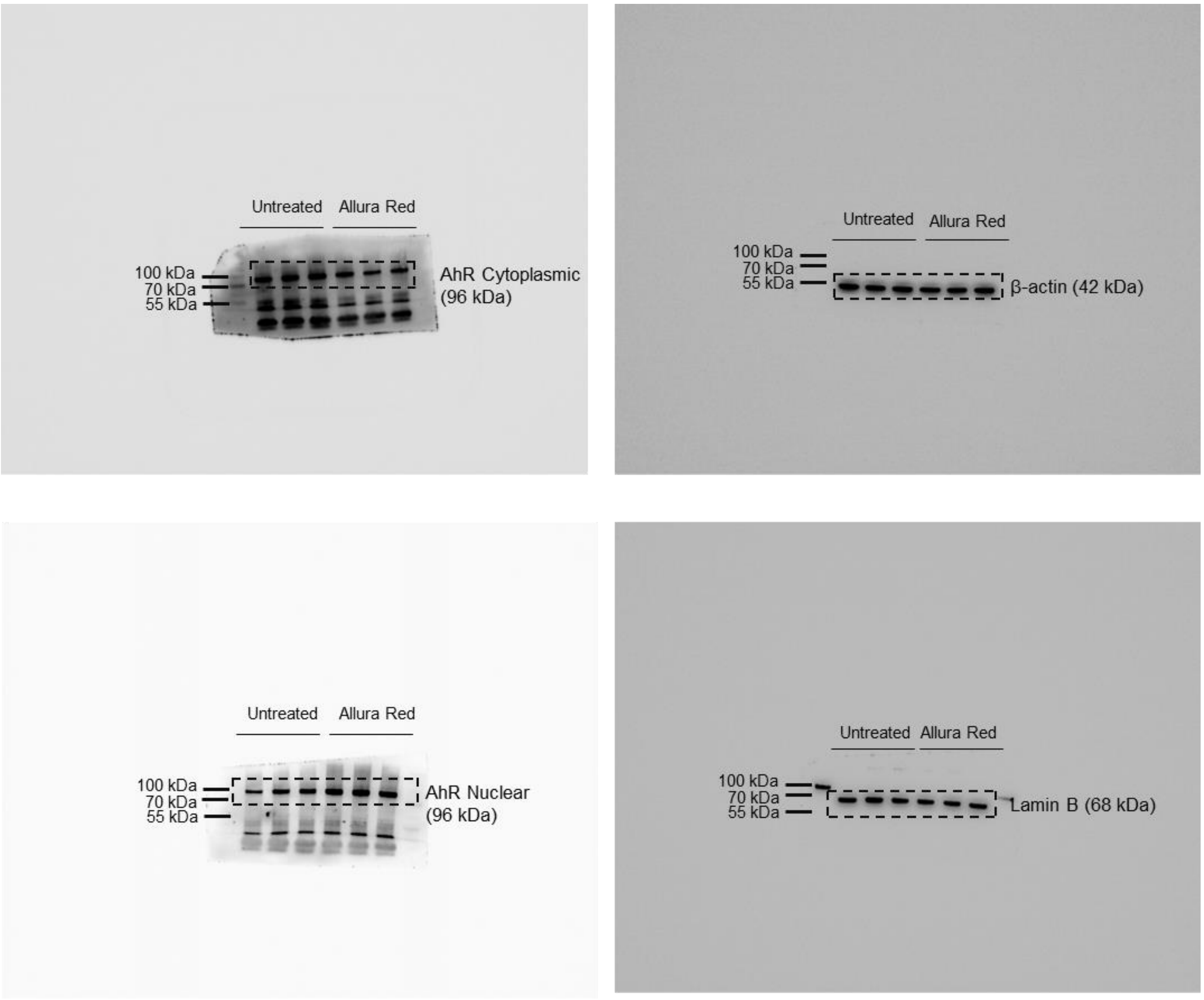

Uncropped blots in Supplementary Figure 9f.

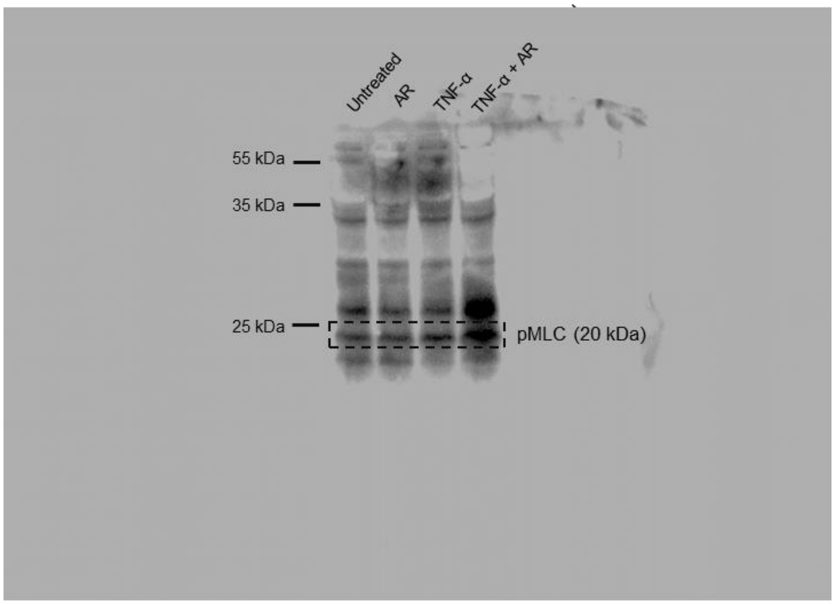

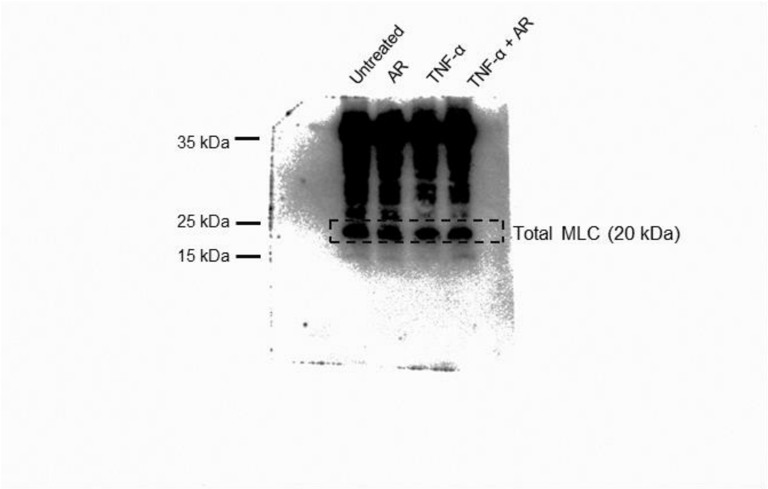

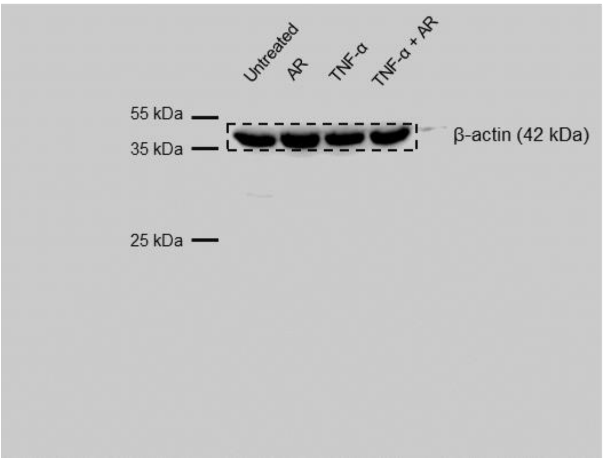

Supplement: Supplementary file 1 — Supplementary Information [file 41467_2022_35309_MOESM1_ESM.pdf]
