# Supplementary material for: Chronic exposure to synthetic food colorant Allura Red AC promotes susceptibility to experimental colitis via intestinal serotonin in mice
Source: Nat Commun. 2022 Dec 20;13:7617. doi: 10.1038/s41467-022-35309-y (PMC9768151; doi:10.1038/s41467-022-35309-y)
Supplement: Supplementary file 2 — Reporting Summary [file 41467_2022_35309_MOESM2_ESM.pdf]

# nature portfolio

Corresponding author(s): Waliul Khan

Last updated by author(s): Dec 10, 2022

# Reporting Summary

Nature Portfolio wishes to improve the reproducibility of the work that we publish. This form provides structure for consistency and transparency in reporting. For further information on Nature Portfolio policies, see our Editorial Policies and the Editorial Policy Checklist.

## Statistics

For all statistical analyses, confirm that the following items are present in the figure legend, table legend, main text, or Methods section.

| n/a | Confirmed | |
|---|---|---|
| ☐ | ☒ | The exact sample size (*n*) for each experimental group/condition, given as a discrete number and unit of measurement |
| ☐ | ☒ | A statement on whether measurements were taken from distinct samples or whether the same sample was measured repeatedly |
| ☐ | ☒ | The statistical test(s) used AND whether they are one- or two-sided<br>*Only common tests should be described solely by name; describe more complex techniques in the Methods section.* |
| ☒ | ☐ | A description of all covariates tested |
| ☐ | ☒ | A description of any assumptions or corrections, such as tests of normality and adjustment for multiple comparisons |
| ☐ | ☒ | A full description of the statistical parameters including central tendency (e.g. means) or other basic estimates (e.g. regression coefficient) AND variation (e.g. standard deviation) or associated estimates of uncertainty (e.g. confidence intervals) |
| ☐ | ☒ | For null hypothesis testing, the test statistic (e.g. *F*, *t*, *r*) with confidence intervals, effect sizes, degrees of freedom and *P* value noted<br>*Give P values as exact values whenever suitable.* |
| ☒ | ☐ | For Bayesian analysis, information on the choice of priors and Markov chain Monte Carlo settings |
| ☒ | ☐ | For hierarchical and complex designs, identification of the appropriate level for tests and full reporting of outcomes |
| ☒ | ☐ | Estimates of effect sizes (e.g. Cohen's *d*, Pearson's *r*), indicating how they were calculated |

*Our web collection on statistics for biologists contains articles on many of the points above.*

## Software and code

Policy information about availability of computer code

| | |
|---|---|
| Data collection | NIS-Elements Basic Research Imaging Software (v 3.2), BD FACSDiva (v 6.1.2). |
| Data analysis | GraphPad Prism (v 5), ImageJ (v 1.4.8), Cutadapt software (v 1.2.1), DADA2 pipeline, phyloseq package. |

For manuscripts utilizing custom algorithms or software that are central to the research but not yet described in published literature, software must be made available to editors and reviewers. We strongly encourage code deposition in a community repository (e.g. GitHub). See the Nature Portfolio guidelines for submitting code & software for further information.

## Data

Policy information about availability of data

All manuscripts must include a data availability statement. This statement should provide the following information, where applicable:

- Accession codes, unique identifiers, or web links for publicly available datasets
- A description of any restrictions on data availability
- For clinical datasets or third party data, please ensure that the statement adheres to our policy

A data availability statement is included in the manuscript. All relevant data generated or analyzed during this study are included in this published article and the supplementary information file. SILVA reference database v 1.2.8 was used for assigning taxonomy. Source data including large csv files are also provided in Source Data File.

## Human research participants

Policy information about studies involving human research participants and Sex and Gender in Research.

| | |
|---|---|
| Reporting on sex and gender | N/A |
| Population characteristics | N/A |
| Recruitment | N/A |
| Ethics oversight | N/A |

Note that full information on the approval of the study protocol must also be provided in the manuscript.

# Field-specific reporting

Please select the one below that is the best fit for your research. If you are not sure, read the appropriate sections before making your selection.

☒ Life sciences ☐ Behavioural & social sciences ☐ Ecological, evolutionary & environmental sciences

For a reference copy of the document with all sections, see nature.com/documents/nr-reporting-summary-flat.pdf

# Life sciences study design

All studies must disclose on these points even when the disclosure is negative.

| | |
|---|---|
| Sample size | As this study was a exploratory study, the sample size was not calculated, but was estimated based on previous experiments performed in the lab using similar models. |
| Data exclusions | Any mice which were severely injured due to fighting was excluded from the study and were not used for analyses. |
| Replication | Replication was performed in all experiments and samples when possible. Multiple cohorts were done for in vivo experiments with enough animal size per group based on previous experiments performed in the lab using similar models. In vitro/ex vivo experiments were replicated with similar results, and specific number of independent in vitro/ex vivo experiments performed during this study is also provided in the figure legends. |
| Randomization | Mice were randomly assigned to groups based on having similar body weight at the beginning of the study and after AR exposure prior to DSS treatment. Randomization is not relevant to in vitro/ex vivo experiments |
| Blinding | Prior to beginning of each in vivo study, the investigators were blinded to group allocation. Investigators were not blinded for in vitro/ex vivo assays and other objective assessments. |

# Reporting for specific materials, systems and methods

We require information from authors about some types of materials, experimental systems and methods used in many studies. Here, indicate whether each material, system or method listed is relevant to your study. If you are not sure if a list item applies to your research, read the appropriate section before selecting a response.

### Materials & experimental systems

| n/a | Involved in the study | |
|---|---|---|
| ☐ | ☒ | Antibodies |
| ☐ | ☒ | Eukaryotic cell lines |
| ☒ | ☐ | Palaeontology and archaeology |
| ☐ | ☒ | Animals and other organisms |
| ☒ | ☐ | Clinical data |
| ☒ | ☐ | Dual use research of concern |

### Methods

| n/a | Involved in the study | |
|---|---|---|
| ☒ | ☐ | ChIP-seq |
| ☐ | ☒ | Flow cytometry |
| ☒ | ☐ | MRI-based neuroimaging |

## Antibodies

| | |
|---|---|
| Antibodies used | PE-cy7-conjugated anti-mouse CD3 (#100219, BioLegend; 1:100), APC-conjugated anti-mouse CD4 (#553051, BD Biosciences; 1:100), and FITC-conjugated anti-mouse CD45 (#553080, BD Biosciences; 1:100), pMLCSer19 (#3671, Cell Signaling Technology; 1:1000), |

total MLC (#3672, Cell Signaling Technology; 1:1000), ZO-1 (#40-2200, Thermo Fisher Scientific; 1:1000 for western blot; 1:100 for immunofluorescence staining), AhR (#sc-133088, Santa Cruz Biotechnology; 1:1000), β-actin (#4970, Cell Signaling Technology; 1:1000), Lamin B1 (ab65986, Abcam; 1:1000), anti-rabbit horseradish peroxidase–linked antibody (#7074, Cell Signaling Technology; 1:5000), anti-mouse horseradish peroxidase–linked antibody (#7076, Cell Signaling Technology; 1:5000), anti-mouse 5-HT (#ab16007, Abcam; 1:100), Alexa Flouor-568 goat anti-mouse IgG (H+L) (#A-11031, Life Technologies; 1:1000), Alexa Fluor-488 donkey anti-rabbit IgG (H+L) (#A-21206, Life Technologies; 1:1000).

Validation

PE-cy7-conjugated anti-mouse CD3, APC-conjugated anti-mouse CD4, and FITC-conjugated anti-mouse CD45 were chosen based on internal optimization data verified by flow cytometry; pMLCSer19 was chosen based on PMID26752649, and total MLC was chosen based on PMID30485810; ZO-1 was chosen based on PMID31244920, and AhR was chosen based on PMID30626868; Anti-rabbit horseradish peroxidase–linked and anti-mouse horseradish peroxidase–linked antibodies were chosen based on PMID34739317; Anti-mouse 5-HT was chosen based on PMID30952815, and Alexa Flouor-568 goat anti-mouse IgG (H+L) and Alexa Fluor-488 donkey anti-rabbit IgG (H+L) were chosen based on PMID26527214 or PMID34739317, respectively. Antibody validation can be found on the respective manufacturers websites, including relevant citations using these antibodies.

## Eukaryotic cell lines

Policy information about cell lines and Sex and Gender in Research

Cell line source(s)

BON cells were a gift from Dr. Milena Bogunovic (The Mount Sinai School of Medicine, New York, USA). HT-29 cells (ATCC HTB-38) were a gift from Dr. Kris Chadee (University of Calgary, Canada). Murine intestinal organoids were cultured by harvesting the whole colonic crypts from male mice.

Authentication

BON cells are routinely used as a model of human EC cell (Siddique et al., Neuroendocrinology, 2009; Reigstad et al., FASEB J., 2015). HT-29 cells are broadly used in the literature as a model of human colonic epithelial cells. As these cell lines were kindly provided by Drs. Bogunovic and Chadee, respectively, none of the cell lines used were authenticated. Murine intestinal organoid culture is broadly used and authenticated in the literature (PMID19329995; PMID21889923).

Mycoplasma contamination

All cell lines tested negative for mycoplasma determination.

Commonly misidentified lines
(See ICLAC register)

No commonly misidentified cell lines were used in this study.

## Animals and other research organisms

Policy information about studies involving animals; ARRIVE guidelines recommended for reporting animal research, and Sex and Gender in Research

Laboratory animals

Male and female germ-free (in-house), C57BL/6 (Taconic), Tph1-/- (in-house; on C57BL/6 background ), SERT-/- (Jackson Laboratories), RAG1-/- (Jackson Laboratories), and C57BL/6 mice (Jackson Laboratories) used in this study were 8-12 weeks old. For early life exposure experiment (Supplementary Figure 7), 4-week-old male mice were used. This information is stated in the figure legends of the published article.

Wild animals

No wild animals were used in this study

Reporting on sex

Sex was not considered in this study.

Field-collected samples

No samples collected from the field

Ethics oversight

All experiments were conducted with approval from the McMaster University Animal Care Committee and McMaster Animal Research Ethics Board (AREB) in an amendment to the Animal Utilization Protocol (AUP: 19-02-09)

Note that full information on the approval of the study protocol must also be provided in the manuscript.

## Flow Cytometry

### Plots

Confirm that:

☒ The axis labels state the marker and fluorochrome used (e.g. CD4-FITC).

☒ The axis scales are clearly visible. Include numbers along axes only for bottom left plot of group (a 'group' is an analysis of identical markers).

☒ All plots are contour plots with outliers or pseudocolor plots.

☒ A numerical value for number of cells or percentage (with statistics) is provided.

### Methodology

Sample preparation

Single cell suspension of healthy spleen derived from C57BL/6J were isolated with mouse naive CD4+ T cell isolation kit, which were then stained with fluorochrome labeled cell-surface antibodies.

| | |
|---|---|
| Instrument | FACS Aria II flow cytometer (BD Biosciences) |
| Software | FACSDiva v 6.1.2 |
| Cell population abundance | The cell population abundance is shown in Supplementary Figure 5. The purity of samples was 94.3%, which was determined by re-running a sorted sample on the same flow cytometer instrument after sort. |
| Gating strategy | The gating strategy is shown in Supplementary Figure 5. Beads used for determining total cell numbers are shown in the FSC and SSC plot. Live CD45RBhi T cells were gated from single cells, from which the CD3 and CD4 population were determined. |

☒ Tick this box to confirm that a figure exemplifying the gating strategy is provided in the Supplementary Information.
